# Complementary Sample Preparation Strategies for Analysis of Cereal β-Glucan Oxidation Products by UPLC-MS/MS

**DOI:** 10.3389/fchem.2017.00090

**Published:** 2017-11-02

**Authors:** Samy Boulos, Laura Nyström

**Affiliations:** Institute of Food, Nutrition and Health, ETH Zurich, Zurich, Switzerland

**Keywords:** β-glucan oxidation, Fenton-reaction, hydrophilic interaction liquid chromatography, lichenase, β-glucosidase, labeling, UPLC, MS/MS

## Abstract

The oxidation of cereal (1→3,1→4)-β-D-glucan can influence the health promoting and technological properties of this linear, soluble homopolysaccharide by introduction of new functional groups or chain scission. Apart from deliberate oxidative modifications, oxidation of β-glucan can already occur during processing and storage, which is mediated by hydroxyl radicals (HO^•^) formed by the Fenton reaction. We present four complementary sample preparation strategies to investigate oat and barley β-glucan oxidation products by hydrophilic interaction ultra-performance liquid chromatography-tandem mass spectrometry (UPLC-MS/MS), employing selective enzymatic digestion, graphitized carbon solid phase extraction (SPE), and functional group labeling techniques. The combination of these methods allows for detection of both lytic (C1, C3/4, C5) and non-lytic (C2, C4/3, C6) oxidation products resulting from HO^•^-attack at different glucose-carbons. By treating oxidized β-glucan with lichenase and β-glucosidase, only oxidized parts of the polymer remained in oligomeric form, which could be separated by SPE from the vast majority of non-oxidized glucose units. This allowed for the detection of oligomers with mid-chain glucuronic acids (C6) and carbonyls, as well as carbonyls at the non-reducing end from lytic C3/C4 oxidation. Neutral reducing ends were detected by reductive amination with anthranilic acid/amide as labeled glucose and cross-ring cleaved units (arabinose, erythrose) after enzyme treatment and SPE. New acidic chain termini were observed by carbodiimide-mediated amidation of carboxylic acids as anilides of gluconic, arabinonic, and erythronic acids. Hence, a full characterization of all types of oxidation products was possible by combining complementary sample preparation strategies. Differences in fine structure depending on source (oat vs. barley) translates to the ratio of observed oxidized oligomers, with in-depth analysis corroborating a random HO^•^-attack on glucose units irrespective of glycosidic linkage and neighborhood. The method was demonstrated to be (1) sufficiently sensitive to allow for the analysis of oxidation products also from a mild ascorbate-driven Fenton reaction, and (2) to be specific for cereal β-glucan even in the presence of other co-oxidized polysaccharides. This opens doors to applications in food processing to assess potential oxidations and provides the detailed structural basis to understand the effect oxidized functional groups have on β-glucan's health promoting and technological properties.

## Introduction

**Figure 1:**
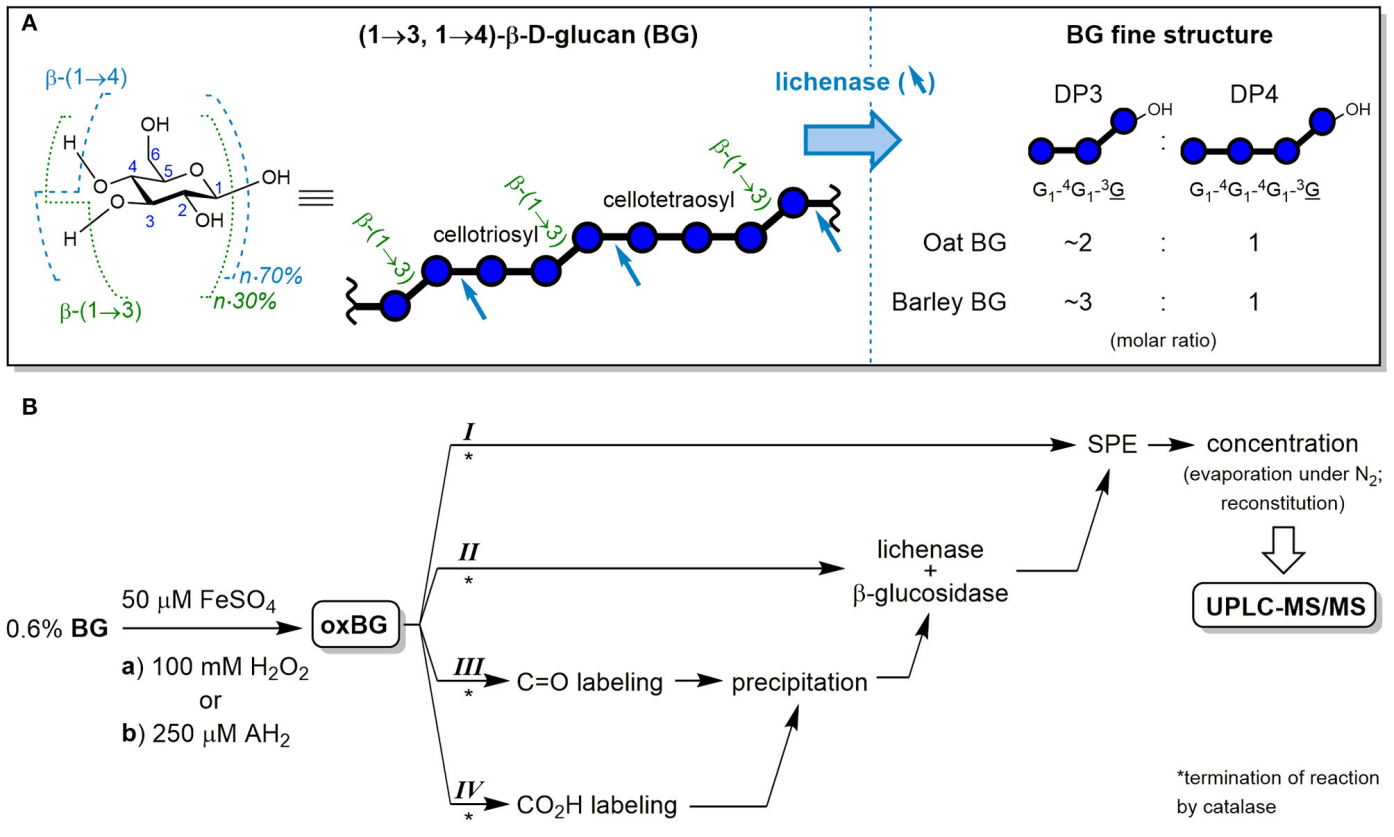
**(A)** Chemical structure of the glucopyranose (Glc) repeating unit of cereal β-D-glucan (BG) with numbered carbons and indicated glycosidic linkage proportions, together with the symbolic representation of the polysaccharide and its cellulosic β-(1→4) regions. In addition, the fine structure analysis by selective hydrolysis with lichenase is shown, with blue arrows indicating the specific β-(1→4)-linkages susceptible to cleavage by the *endo*-enzyme and the resulting characteristic ratios of formed oligosaccharides DP3 and DP4. **(B)** Workflow of the cereal BG oxidation study (from oat and barley) with the two oxidation conditions (a) and (b) and the four complementary sample preparation strategies I–IV. Blue ○ = G, glucose unit; blue ○–OH = G, reducing end glucose unit; oxBG; oxidized β-glucan; AH_2_, ascorbic acid; C=O, carbonyl group; CO_2_H, carboxylic acid group; SPE, solid phase extraction; UPLC-MS/MS, ultraperformance liquid chromatography tandem mass spectrometry.

Cereal mixed-linkage (1→3,1→4)-β-d-glucan (BG) is a soluble dietary fiber with great potential for functional foods due to its well-established health-promoting properties such as blood cholesterol lowering and blood glucose regulation (FDA, 2009; EFSA, 2011). BG is mainly (≥90%) comprised of cellotriosyl and cellotetraosyl units linked by β-(1→3) glycosidic bonds, forming a linear homo-polysaccharide of β-d-glucopyranose (see Figure 1A). The cellotriosyl/cellotetraosyl ratio is characteristic for the source of BG, with oat (OBG; 1.7–2.4) having smaller ratios than barley (BBG; 2.7–3.6), and is typically determined by hydrolysis with lichenase and ion-exchange chromatography-pulsed amperometric detection. The *endo*-enzyme lichenase (EC 3.2.1.73) selectively cleaves the β-(1→4)-linkages of β-(1→3)-linked glucose units, releasing gluco-oligomers (Glc_*n*_) with β-(1→3)-linked reducing end units (abbreviated as G_1_-^4^G_1_-^3^G and G_1_-^4^G_1_-^4^G_1_-^3^G for degrees of polymerization (DP) of *n* = 3 and 4, respectively; see Figure 1A). The DP3/DP4 fine structure differences have an impact on BG's physico-chemical properties, for example, BBG having a higher propensity to form gels than OBG (Tosh et al., 2004; Lazaridou and Biliaderis, 2007; Wood, 2010).

During processing and storage, BG can be degraded enzymatically or chemically to lower-molecular-weight products with diminished viscosity (Tosh et al., 2010) and health benefits (Regand et al., 2009). Thereby, the chemically-induced oxidative degradation of BG has been shown to occur in the presence of substances commonly found in foodstuff when in contact to atmospheric oxygen (O_2_), namely traces of transition metal (Fe or Cu) and a reducing agent such as ascorbic acid (AH_2_) (Schuchmann and Sonntag, 1978; Kivelä et al., 2009a,b). For example, during thermal or high pressure treatment of BG solutions, new carbonyl groups of up to ~10 μmol/g BG (or ~2 C=O per 1,000 repeating units) were detected (Kivelä et al., 2012). The reactive oxygen species responsible for the loss in viscosity/ molecular weight (*M*_*w*_) was identified by indirect spin trapping and electron spin resonance (ESR) spectroscopy as hydroxyl radical (HO^•^; Faure et al., 2013b). The catalytic cycle of HO^•^ production is thought to be induced by the pro-oxidant activity of AH_2_, which reduces intrinsic iron and dissolved O_2_ to produce Fe^2+^ and hydrogen peroxide (H_2_O_2_; Michels and Frei, 2013), the two substrates for the Fenton reaction (1894):

(1) $$\begin{array}{r} {\text{Fe}}^{2+}+{\text{H}}_{2}{\text{O}}_{2}\to {\text{Fe}}^{3+}+{\text{OH}}^{-}+{\text{HO}}^{•}. \end{array}$$

Apart from threatening the molecular integrity of BG and its health benefits (Wood et al., 2000; Wolever et al., 2010; Regand et al., 2011), deliberate incorporation of new functional groups through oxidation has also been reported to change BG's technological (Lee et al., 2011) and health promoting properties (Park et al., 2009), providing great potential to influence BG functionality. Hence, cereal BG oxidation has been the focus of numerous studies employing various analytical techniques to investigate the radical reaction (ESR), to characterize the products (NMR/FT-IR), and monosaccharide composition (HPAEC-PAD), to quantify carbonyl or carboxylate groups (titration, fluorescent labeling), and to determine the change in bulk properties (rheology, *M*_*w*_ distribution, aggregation; Kivelä et al., 2010, 2011, 2012; Faure et al., 2012, 2013a, 2014; Mäkelä et al., 2015, 2017). On a molecular level, indirect detection of oxo-products from HO^•^-mediated degradation of barley BG was reported by Iurlaro et al. (2014), employing reductive tritium labeling followed by enzymatic or acid catalyzed hydrolysis and analysis by paper- and thin-layer chromatography. They confirmed the formation of new reducing ends and mid-chain oxo-groups, allowing to some extent the localization of the oxidative changes on monosaccharide units, but providing little information about their original connectivity prior to hydrolysis and no information about carboxylic acid products. In other fields of polysaccharide degradation, a recent notable accomplishment was the detection of HO^•^-attack in ripening fruit with a sensitive method developed by Airianah et al. (2016) for pectin oxidation using labeling of carbonyl groups by reductive amination, enzymatic digestion and electrophoresis/HPLC with fluorescent detection (Vreeburg et al., 2014).

For a deeper understanding on a molecular level, we have recently studied BG oxidation with constitutionally isomeric oligosaccharide model compounds by means of hydrophilic interaction ultra-performance liquid chromatography tandem mass spectrometry (UPLC-MS/MS) with high resolution detection (Boulos and Nyström, 2016). The direct study of polysaccharide oxidation by MS techniques, on the other hand, is impeded by the large *M*_*w*_ and comparatively low numbers of oxidation sites along the polymer chain. In the literature, various strategies have been used to study the oxidation of other polysaccharides by MS or LC-MS, such as enzymatic treatments to remove non-oxidized portions as in the lytic polysaccharide monooxygenase (LPMO)-induced degradation of cellulose (Westereng et al., 2016) and the HO^•^-mediated oxidation of starch (Simões et al., 2016), functional group labeling with laminaran oxidation for higher sensitivity/ LC-separation (Ovalle et al., 2001), and analysis of released oligomers (directly or after carbonyl reduction/ tagging) with hyaluronan (Zhao et al., 2013) and pneumococcal type-3 polysaccharide (Li et al., 2015). Typically, harsh oxidative conditions were applied in those studies to accumulate enough products, presumably leading to secondary oxidation. However, detection of products from Fenton-induced oxidation of cereal BG polymer specifically, and from mild oxidation of any polysaccharide in general (<1% modified repeating units), have yet to be accomplished by LC-MS.

In this paper, we used UPLC-MS/MS to investigate the Fenton-induced degradation of polymeric BG. The aim was to identify oxidation products specific to the mixed-linkage nature of cereal BG, to document differences in product profiles depending on its source (oat vs. barley) and shed light on the degradation mechanism. For that purpose, we developed analytical procedures sensitive enough for detection of primary products formed in a mild oxidation relevant to foodstuff and food processing/storage: the ascorbate-driven Fenton reaction. We present four complementary sample preparation strategies (I–IV) to obtain a complete picture of BG oxidation by means of UPLC-MS/MS, employing solid phase extraction (SPE), enzymatic digestions, and functional group labeling, allowing for specific detection of cereal BG oxidation even in the presence of other glucose-polymers (see Figure 1B).

## Materials and methods

### Materials

High viscosity barley β-glucan (BBG; >94% dry weight basis; 495 kg/mol; Lot 90501b) and high viscosity oat β-glucan (OBG; >94% dry weight basis; 361 kg/mol; Lot 80608c) were purchased from Megazyme (Ireland) and as 1% aqueous solutions, purified by extensive dialysis against EDTA (1 mM) and water, followed by precipitation with two volumes of ethanol, centrifugation, lyophilization, and milling. Stock solutions of 1.0% (m/v) OBG and BBG were prepared by dissolving the purified samples in water under stirring and heating in a boiling water bath for 2 h with occasional vigorous shaking. d-Laminaribiose (G_1_-^3^G), 3-*O*-β-cellobiosyl-d-glucose (G_1_-^4^G_1_-^3^G), and 3-*O*-β-cellotriosyl-d-glucose (G_1_-^4^G_1_-^4^G_1_-^3^G) were purchased from Megazyme (Ireland) with a purity of >95%, as well as six cereal β-glucan molecular weight standards, lichenase (from *Bacillus subtilis*; EC 3.2.1.73; GH family 16) and β-glucosidase (from *Aspergillus niger*; EC 3.2.1.21; GH family 3). All other chemicals were of analytical purity and used without further purification unless otherwise noted. d-(+)-Cellobiose (G_1_-^4^G), anhydrous d-(+)-glucose (Glc), 35% hydrogen peroxide (H_2_O_2_), sodium hydroxide (NaOH), iron(II) sulfate heptahydrate (FeSO_4_×7H_2_O), 25% aqueous ammonia (NH_3_) and formic acid (both LC-MS grade), ammonium formate (NH_4_HCO_2_; ≥99.995% trace metal basis), anhydrous sodium acetate (NaOAc), sodium dihydrogen phosphate, sodium nitrate (NaNO_3_), sodium azide (NaN_3_), methanol, 2-propanol, acetone, 2-aminobenzamide (2-AB), sodium cyanoborohydride (NaBH_3_CN; 95%), aniline (PhNH_2_), 1-ethyl-3-(3-dimethylaminopropyl)-carbodiimide hydrochloride (EDC·HCl), 37% hydrochloric acid (HCl), and catalase from bovine (11,000 U/mg) were purchased from Sigma-Aldrich Chemie GmbH (Germany). l-Ascorbic acid (AH_2_) was purchased from Fluka (Germany). Anthranilic acid (2-AA; 2-aminobenzoic acid) was prepared by simple hydrolysis of 2-aminobenzamide with NaOH at 100°C and precipitation with HCl (pH 3.5). Corn starch was purchased at the local supermarket at Migros, Switzerland [Patissier brand; specified composition (not dried): 88% carbohydrates, 0.6% fat, <0.5% proteins, 0% fibers], and 2% (m/v) stock solutions/suspensions prepared as described above for OBG/BBG. Acetonitrile (ACN; ULC/MS grade) was from Biosolve BV (the Netherlands) and aqueous solutions were prepared with nanopure Milli-Q® water (H_2_O; ≥18.2 MΩ·cm at 25°C). Unless otherwise noted, all solutions refer to aqueous solutions, and mixtures of liquids (e.g., solvents) are indicated by ratio or percentage in terms of volume (v/v).

### Size exclusion chromatography (SEC)

The weight-average molar masses (*M*_*w*_) of the BG samples were determined with a chromatographic system composed of binary pump, degasser, thermostated column compartment, and autosampler, all from HP (Series 1100, Hewlett Packard, USA). A pre-column (Viscotek AGuard Col. 50 × 6 mm, Malvern Instruments Ltd, United Kingdom) was used together with an A5000 column (Viscotek, 300 × 7.8 mm, Malvern Instruments Ltd, United Kingdom) and a Suprema 30,000 column (10 μm, 300 × 8 mm, PSS Polymer Standards Service GmbH, Germany). As eluent, aqueous 0.1 M NaNO_3_ with 0.02% NaN_3_ was used after filtration (0.45 μm). The columns were maintained at 35°C and the flow rate was set at 1 mL/min. The injection volume was 50 μL and the elution was recorded with a refractive index detector (Series 1200, Agilent Technologies AG, Switzerland). Values were calculated using ChemStation software (ChemStation for LC 3D systems, Rev B.04.02 SP1) with the Cirrus GPC/SEC software add-on (version 3.4.1), both from Agilent. Six BG *M*_*w*_ standards (0.1% in eluent) were used to generate a calibration curve with reported peak molecular weight (*M*_*p*_) of 33.6–667 kg/mol. Peak band broadening was corrected according to Malawer and Senak (2003), employing calculated symmetric band broadening and skewing factors as a function of elution time on the basis of reported *M*_*w*_ and *M*_*n*_ of injected native, undialyzed β-glucans (Megazyme, Ireland).

### Oxidation of BG

Two reaction conditions for OBG and BBG oxidation were tested in triplicates as aqueous solutions (10 mL) in Falcon tubes, both containing BG (0.6% (m/v), corresponding to 37 mM of total anhydroglucose repeating units) and FeSO_4_ (50 μM). Degradation was induced by either (a) H_2_O_2_ (100 mM; harsh conditions) for method development or (b) AH_2_ (250 μM; mild conditions) to mimic conditions more relevant to food. The reaction mixtures were kept in the dark at room temperature for 24 h with access to air. As the control, aqueous solutions of BG (0.6%) were prepared without addition of reagents. After the degradation, the solutions were treated with catalase and phosphate buffer pH 6.5 (25 U/mL and 10 mM final concentrations, respectively) to eliminate excess H_2_O_2_ and/or prevent further oxidation, and stored at 4°C until subjection to sample preparation strategies I–IV (within 1 week; see Figure 1B for overview).

### Strategy I: solid phase extraction (SPE)

Released oligomers from the oxidation of BG were separated from polymeric BG by a graphitized non-porous carbon SPE method adapted from a procedure by Packer et al. (1998) for desalting oligosaccharide solutions. In short, Supelclean™ ENVI-Carb SPE cartridges (3 mL, 0.25 g) from SUPELCO® (Sigma-Aldrich, Germany) were preconditioned with ACN (3 mL) and H_2_O (3 mL). An aliquot of the BG oxidation mixture or control (2.00 mL, 0.6%) was directly loaded on the SPE unit and the cartridge washed with H_2_O (1 × 3, 2 × 1.5 mL), both with a drop rate of 1–3 drops per second. The neutral and acidic oligomeric products were eluted with 0.1% formic acid in 1:1 ACN/H_2_O (2.5 mL) and analyzed by UPLC-MS/MS after evaporation (stream of N_2_, 40°C)/reconstitution in 1:1 ACN/H_2_O (100 μL).

### Strategy II: enzyme treatment

Enzyme digestion was based on the AOAC official method 995.16 for BG quantification with adjustments to make it suitable for our purposes. An aliquot of catalase treated, oxidized, or control BG solution (2.00 mL, 0.6%, 74 μmol total monosaccharide units) in a Falcon tube was subjected to lichenase (200 μL, 20 U/mL, 4 U) and phosphate buffer (40 μL, 1 M, pH 6.5) at 37°C under shaking for 3 h, followed by overnight β-glucosidase digestion (50 μL, 20 U/mL, 1 U) with addition of acetate buffer (250 μL, 0.5 M, pH 4.0) at room temperature, resulting in a pH 4–4.5. Excess enzyme loadings were chosen to maximize hydrolysis of all lichenase- and β-glucosidase-susceptible sites along the BG backbone also in the proximity of new functional groups that might hinder the digestion. The resulting oligomeric oxidation products were fractionized by SPE by loading the whole digested sample as in the procedure described above (strategy I). The collected fractions eluted with 0.1% formic acid in 3:1 ACN/H_2_O (2.5 mL) were directly analyzed by UPLC-MS/MS unless otherwise noted.

### Strategy III: carbonyl labeling

Carbonyls in the oxidized BG were labeled by reductive amination using NaBH_3_CN and an aromatic amine, namely 2-aminobenzamide (2-AB) or anthranilic acid (2-AA; 2-aminobenzoic acid).

#### 2-Aminobenzamide (2-AB)

Labeling of BG with 2-AB was loosely based on a procedure of Bigge et al. (1995), but in aqueous methanol instead of DMSO as in the method reported by Anumula and Dhume (1998). To an aliquot of catalase treated oxidized or control BG solution (300 μL, 0.6%, 11 μmol total monosaccharide units) in a Falcon tube were added AcOH (120 μL, 2,100 μmol), aqueous NaBH_3_CN (120 μL, 1.4 M, 168 μmol), and 2-AB (450 μL, 0.8 M in aq. 50% MeOH, 360 μmol). The closed tube was vortex mixed and heated to 80°C for 10 min, followed by addition of more NaBH_3_CN (120 μL, 1.4 M, 168 μmol), mixing, and storage in the dark overnight at room temperature. To remove excess reagents, the labeled BG was precipitated with two volumes of 2-propanol and four volumes of ACN, mixed, and the suspension centrifuged (4,000 rpm, 15 min, 4°C). The supernatant was discarded and the pellet re-suspended in 95:5 ACN/H_2_O (10 mL), centrifuged, and decanted. This process was repeated once with MeOH (10 mL). The resulting pellet was dissolved/suspended in H_2_O (2 mL) and treated with lichenase and β-glucosidase as described in the enzyme treatment above (strategy II). SPE cartridges were conditioned as described above, and the labeled products eluted with 0.1% formic acid in 1:3 ACN/H_2_O (2.5 mL, fraction 1) and 0.1% formic acid in 1:1 ACN/H_2_O (1.5 mL, fraction 2). The SPE fractions were analyzed by UPLC-MS/MS after evaporation/ reconstitution of an aliquot (1 mL) in 1:1 ACN/H_2_O (100 μL).

#### Anthranilic acid (2-AA)

Labeling with 2-AA was based on the method reported by Huang et al. (2000) for polysaccharide substrates and was under milder conditions. In short, to an HPLC-vial containing solid 2-AA (8.2 mg, 60 μmol) and NaBH_3_CN (19 mg, 300 μmol) was added an aliquot of catalase treated, oxidized or control BG solution (300 μL, 0.6%). The vial was firmly closed and the suspension heated to 65°C in a water bath for 2 h with vigorous shaking every 5 min in the first half hour for 2-AA to fully dissolve. After cooling to room temperature, the labeled BG was purified by repeated precipitation, digested with enzymes, and fractionized by SPE as described above for the 2-AB labeling, but with a higher volume for fraction 2 (3 mL), and the SPE fractions analyzed by UPLC-MS.

### Strategy IV: carboxylic acid labeling

The carboxylic acids of the oxidized BG were labeled by amidation using EDC-mediated coupling with aniline (PhNH_2_) based on a procedure by Yang et al. (2008). The label stock solution was prepared with addition of HCl to adjust its pH to 4.5. To an aliquot of catalase treated, oxidized or control BG solution (300 μL, 0.6%, 11 μmol total monosaccharide units), the stock solution of PhNH_2_ (600 μL, 0.5 M in 20% MeOH, 300 μmol) was added. The condensation was induced by addition of aqueous EDC·HCl (100 μL, 1 M, 100 μmol), the reaction mixture was vortex mixed and stored in the dark at room temperature for 16 h. To remove excess reagents, the labeled BG was precipitated, digested with enzymes, and fractionized by SPE as described above for the carbonyl labeling (strategy III), but with two adjustments: an additional resuspension step with acetone (10 mL) before the final MeOH washing, and SPE elution directly with 0.1% formic acid in 1:1 ACN/H_2_O (3 mL).

### Co-oxidation of BG and starch

In order to demonstrate the specific detection of BG oxidation products in the presence of other glucose polymers, a reaction mixture containing BBG (0.6%), corn starch (0.6%), H_2_O_2_ (100 mM), and FeSO_4_ (50 μM) was prepared (5 mL total volume) and allowed to react at room temperature for 24 h with access to air. A solution of BG (0.6%) and starch (0.6%) without reagents was used as the blank. As a control, starch (0.6%) was oxidized alone (100 mM H_2_O_2_, 50 μM FeSO_4_). Aliquots of the reaction mixture (2 mL), of the blank and the control were subjected to sample preparation strategy II (enzyme digestion/SPE) as described above and analyzed by UPLC-MS/MS.

### UPLC-MS/MS analysis

A Waters Acquity UPLC system equipped with an Acquity UPLC BEH Amide column (2.1 × 150 mm, 1.7 μm) was used unless otherwise noted, coupled to a Synapt G2 MS system composed of an electrospray ionization (ESI) source and a quadrupole time-of-flight (qToF) analyzer (Waters Corp., Milford, MA, USA) as described earlier (Boulos and Nyström, 2016). The MS was calibrated with a sodium formate solution with leucine-enkephalin as the lock mass, and run in negative ion mode unless otherwise noted in the text.

#### Chromatographic conditions

ACN and H_2_O were used as eluents. To avoid strong solvent effects during injection, 8:2 and 2:8 mixtures of ACN/H_2_O were used as weak and strong needle wash solutions, respectively. Injections (2.5 μL) were made in partial loop mode and the column maintained at 35°C with a flow rate of 0.34 mL/min. The gradient started with 20% H_2_O and 80% ACN (both with 0.1% NH_3_ additive), was linearly increased to 40% H_2_O in 12 min (to 50% H_2_O in the case of strategy I to ensure elution of larger oligomers DP > 5), back to the initial conditions in 0.75 min, and reequilibration for 2.25 min (“NH_3_ eluent system”).

For separation of the acidic products, a “buffered eluent system” based on Westereng et al. (2013) was used with modifications (constant buffer concentration to prevent increasing ion suppression). Eluent A and B were composed of ACN/H_2_O 1:3 and 3:1, respectively, both with 60 mM ammonium formate buffer (NH_4_HCO_2_; pH 8). The gradient started isocratically with 0% A, 100% B for 2 min (= 25% H_2_O), was linearly increased to 34% A for 5.5 min (= 42% H_2_O), maintained there for 1.5 min, brought back to 0% A in 1 min, and reequilibrated for 2.5 min.

#### MS conditions

The spectra were acquired in resolution mode and the voltages of capillary, sample cone, and extraction cone were set for the NH_3_ eluent system at 2,000, 15, and 3 V, respectively; higher voltages were used for the buffered ammonium formate eluent (2,000, 25, and 4 V, respectively). The desolvation gas flow rate was 850 L/h at 350°C. The cone gas flow was 20 L/h and the source temperature 120°C. Full scan mass spectra were acquired from *m*/*z* 50 to 1,200 at a scan rate of 1 Hz in centroid mode (test-wise up to *m*/*z* 1,500 to check for DP > 7). Besides the full scan, exact masses were selected to perform MS/MS through collision-induced dissociation (CID) with Argon as collision gas. The low mass (LM) resolution was set to 15 for a very narrow *m*/*z* isolation width of the selected ions, and the transfer collision energies were ramps ranging from 5–10 to 10–60 V depending on propensity for fragmentation. All data were collected and processed using MassLynx software, version 4.1 (Waters Corp., Milford, MA, USA). MS spectra were background-corrected by subtracting the signals between 0 and 1 min of their respective chromatograms, and base peak ion chromatograms (BPI) were background-corrected by subtracting a blank 0 μL injection if not specified otherwise. For integration of peaks, extracted ion chromatograms (XIC) were used with *m*/*z* of the analyte in question ±0.05 Da.

## Results and discussion

**Figure 2:**
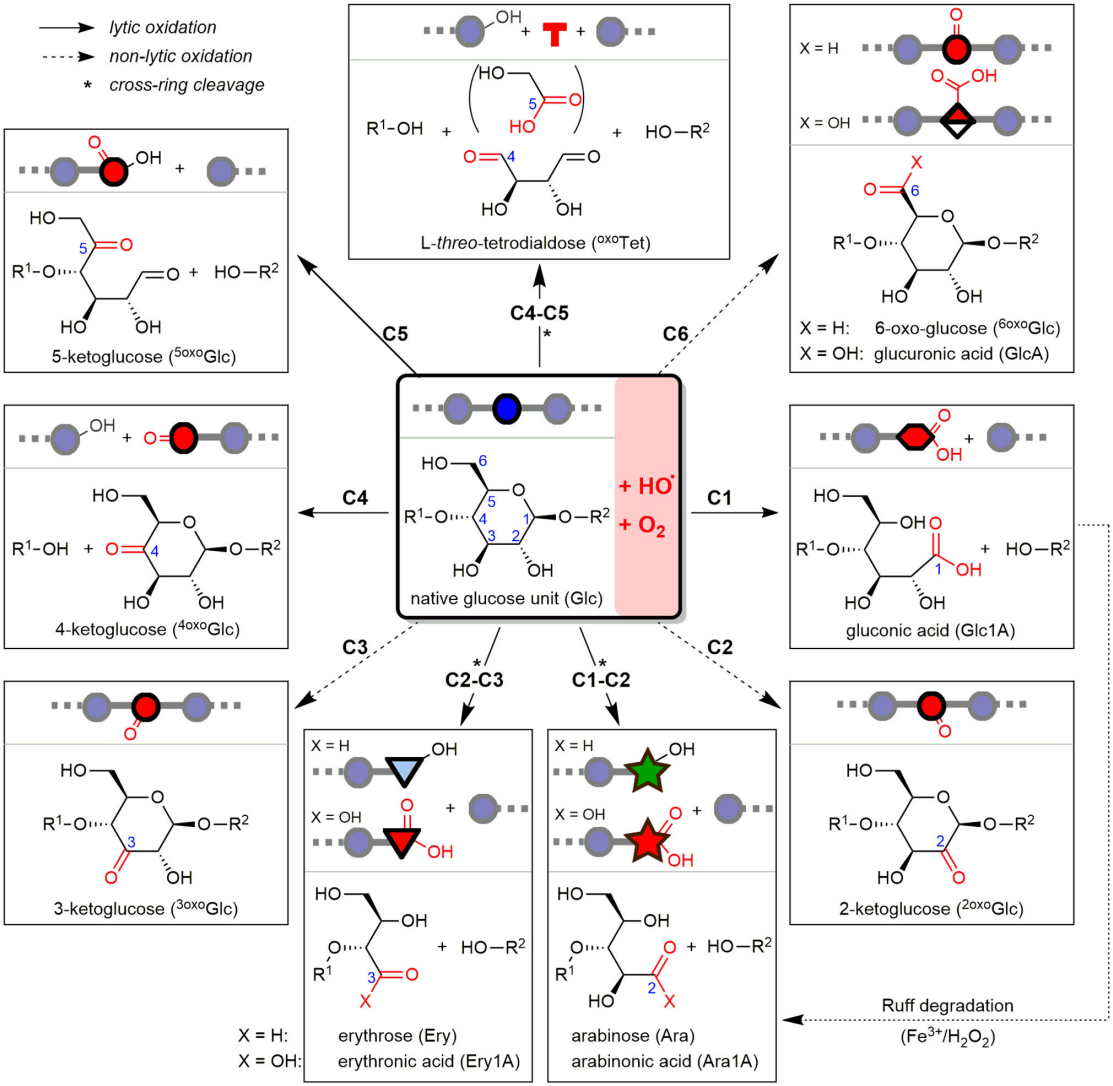
Starting from β-(1→4)-linked glucosyl repeating unit (middle), the detectable oxidation products from hydroxyl radical (HO^•^)-attack at any of the C1–6 glucose carbons after reacting with oxygen (O_2_) are represented as structures, depicted symbols, abbreviations, and full names. Depending on site of attack, the resulting product is formed with or without chain scission, classified as lytic (plane arrows) or non-lytic oxidation (hashed arrows), respectively. Arabinose can by formed both directly under lytic cross-ring cleavage (C1-C2), or as secondary product of gluconic acid (from C1-oxidation) by the indicated, so-called Ruff-degradation (bottom right). ^*^Lytic oxidations under cross-ring cleavage and loss of one or two carbons (e.g., formic acid, glycolic acid).

Oat and barley β-glucan (OBG & BBG) were oxidized by an iron-catalyzed (50 μM FeSO_4_), HO^•^-mediated degradation, either mildly with an ascorbate-driven Fenton reaction (250 μM AH_2_), or harshly with Fenton-induced oxidation using H_2_O_2_ directly (100 mM). To minimize factors that influence the degradation such as potential iron (Faure et al., 2013a) and phytic acid contaminations (Faure et al., 2012; Wang et al., 2017), commercial high viscosity OBG & BBG (>97%) were first dialyzed against EDTA and H_2_O. The oxidized β-glucans were then subjected to four sample preparation strategies I–IV and UPLC-MS/MS analysis (section Part 1—Methodology), which revealed their product profile dependence on β-glucan source (oat vs. barley) and reaction conditions (AH_2_ vs. H_2_O_2_) (section Part 2—Application). At the heart of this study are the major lytic and non-lytic oxidation products that are formed depending on site of HO^•^-attack on glucose carbons C1–6 along the β-glucan backbone. These expected products based on our previous BG oligomer study are shown in Figure 2 as structures, names, abbreviations, and symbolic representations to serve as an overview (Boulos and Nyström, 2016).

### Part 1—methodology

#### SPE strategy I: released oligomers

The first sample preparation strategy entailed running the reaction mixture directly through a non-porous graphitized carbon SPE followed by a washing step with water. As confirmed with control SPE experiments of native BBG solutions spiked with a “glucose ladder” (Glc_*n*_, *n* = 1–6) and TLC, neutral monosaccharides as well as polymeric materials were not retained (see Figure S1 for details). While neutral monosaccharides are known to be washed off with water, the lack of retention for the polysaccharide on graphitized carbon is unexpected in light of adsorption strength being a function of degree of polymerization (DP), at least for dextrans of DP ≤ 24 (Redmond and Packer, 1999). Packer et al. (1998) observed that sodium dodecyl sulfate was retained, unless it formed micelles under certain conditions that could be washed off with water from the carbon solid phase. One may speculate that BG as a hydrocolloid forms supramolecular aggregates that behave similarly. Hence, carbon SPE allowed for isolation of both neutral and acidic oligosaccharides released through harsh oxidation of BG (100 mM H_2_O_2_), which accumulated on the solid phase as expected and could be detected after elution, giving rise to similarly complex UPLC-MS patterns as observed in our previous oligo-BG oxidation study (Boulos and Nyström, 2016).

**Figure 3:**
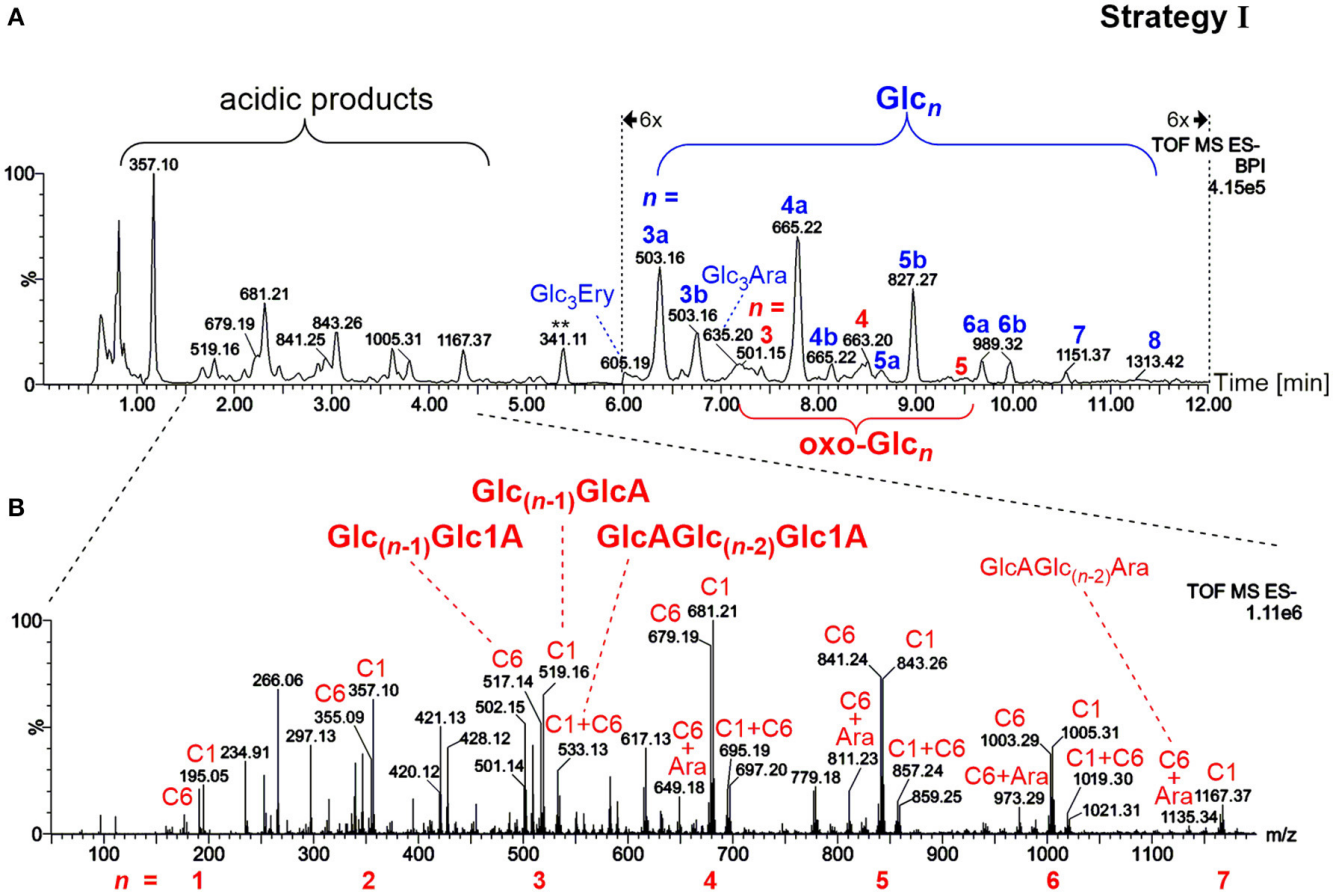
**(A)** UPLC-MS base peak chromatogram in the negative ionization mode using a BEH amide column and ACN/H_2_O gradient (0.1% NH_3_) for analysis of the released oligosaccharides from barley β-glucan (BBG) oxidation (harsh conditions) extracted by SPE and concentrated (sample preparation strategy I). Note the 6x zoom for region 6–12 min. Peaks are labeled with their respective base peak *m/z* and with *n* = number of monosaccharide units in blue for gluco-oligosaccharides Glc_*n*_ and in red for oxo-Glc_*n*_ species (with letter qualifier if same *n* occurs more than once, e.g., 3a, 3b…). **(B)** MS spectrum of the acidic products obtained from the indicated chromatogram region in **(A)**. Signals are labeled with *m/z*, with the glucose-carbon location of the carboxylic acid (C1, C6, or C1+C6), and with type of reducing end if cross-ring cleaved to arabinose (Ara). Glc, glucose; oxo-Glc_*n*_, oligomer Glc_*n*_ oxidized anywhere along the chain (new C=O); Glc_3_Ara/Ery, glucotriose linked to arabinose/erythrose end group; Glc1A, gluconic acid group (C1 oxidized); GlcA, glucuronic acid group (C6 oxidized).

The most prominent detected neutral products as evident in Figure 3A were gluco-oligosaccharides (Glc_*n*_ in blue) and their respective counterparts with one oxidized hydroxyl group (oxo-Glc_*n*_ in red, referring to gluco-oligomers with the carbonyl group located on any of the units/ possible glucose carbons). Isomeric mixtures of mixed-linkage oligomers were detected with random position of the β-(1→3)-linkage along the chain as suggested by MS/MS analysis (Figure S2), in accordance with a random chain scission by HO^•^ irrespective of the linkage type. Acidic products, on the other hand, exhibited as expected low retention under the basic chromatographic conditions (Leijdekkers et al., 2011; Boulos and Nyström, 2016), and could be classified into two groups: (1) oligomers with oxidized end groups, mostly gluconic acids Glc_(*n*−1)_Glc1A from lytic C1-oxidation, and (2) oligomers from C6-oxidation, Glc_(*n*−1)_GlcA (see Figure 3B for MS spectrum). Products carrying carboxyl groups at both C1+C6 positions could also be detected [GlcAGlc_(n−2)_Glc1A], as well as the respective cross-ring cleavage product arabinose (Ara) at the reducing end (GlcAGlc_(n−2)_Ara; for overview of main oxidation product structures, see Figure 2).

It is the nature of random polymer chain oxidations that harsh degradation conditions are required to produce appreciable amounts of small oligomers (*n* = 2–8). Due to the low concentration of the small fraction of products that have a suitable size for UPLC-MS analysis, the sensitivity of this sample preparation strategy is intrinsically low. Indeed, no BG oxidation products could be observed in the mild oxidation (250 μM AH_2_) with this direct SPE method, presumably due to the low degree of oxidation resulting in polymeric products that were too large to be retained by SPE. In fact, estimation of the relative amount of released and SPE collected BG material by phenol-sulfuric acid assay (Saha and Brewer, 1994) indicates that the oligomers with *n* = 2–8 make up <1% of total BG even under the harsh oxidation conditions (100 mM H_2_O_2_). While this SPE strategy allows for the direct analysis of oligomeric oxidation products in their intact form without prior derivatization or hydrolysis, it showcases the necessity for other sample preparation strategies with higher efficiency and access to oxidation sites representative of the whole polymeric material.

#### Enzyme digestion strategy II: polymeric oxidation products

**Figure 4:**
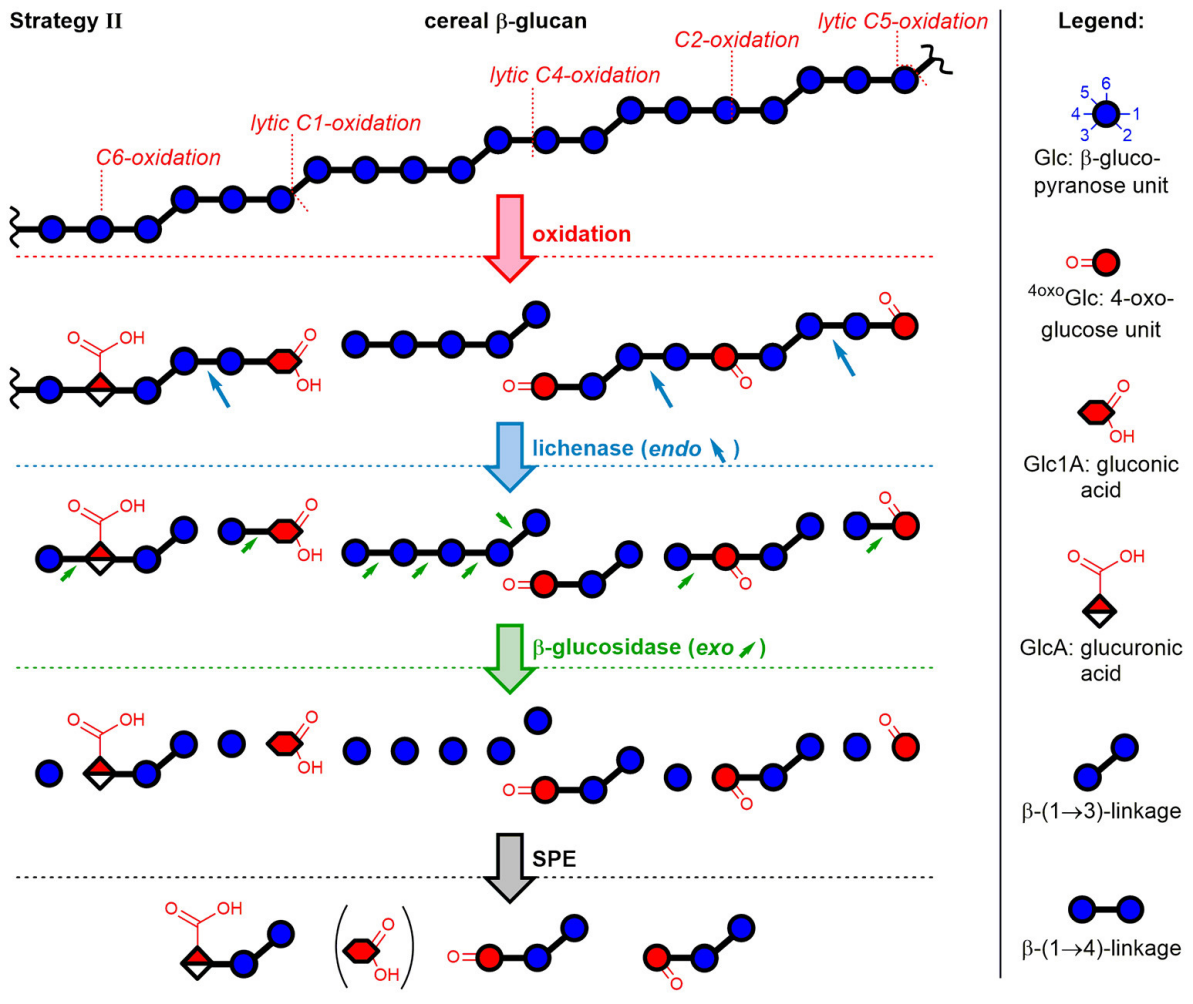
Depiction of mixed-linkage BG and examples of possible oxidation products formed through attack of HO^•^ at different glucose carbons, followed by sample preparation strategy II. Lichenase selectively hydrolyzed as expected the β-(1→4)-linkage of the β-(1→3)-linked glucose units. Digestion by β-glucosidase, an *exo*-enzyme, was found to successively cleave off glucose units, leaving oxidized oligomers behind, whereas native regions largely hydrolyzed to glucose. Graphitized carbon SPE removed monosaccharides from the reaction mixture (acidic monomers only partially removed). Downstream (reducing) end of oligomers is on the right side (symbolic differentiation as ○–OH omitted for clarity).

The second method included enzymatic digestion of the polymer after oxidation and before SPE fractionation, thereby expanding the access of information to the polymeric oxidation products. The strategy revealed which newly introduced functional groups hinder the enzymes from completely hydrolyzing the polymer to monosaccharides. As illustrated in Figure 4 with examples of five oxidation sites on BG, lichenase treatment selectively cleaved as expected (blue arrows) the oxidized BG to give rise to oligomers with a β-(1→3)-linked reducing end unit (for chemical structures, see Figure 2). Subsequent hydrolysis by *exo*-β-glucosidase, which turned out to be more sensitive to steric disturbances, cleaved off native glucose units one by one starting from the non-reducing end on, until it arrived at a modified sugar unit which hindered the enzyme to proceed further. The inability of β-glucosidase to fully hydrolyze oligomers with oxidized non-reducing end units was also observed by Westereng et al. in the case of a 4-oxo/geminal diol group on cello-oligomers (Westereng et al., 2016). The same graphitized carbon SPE as in sample preparation strategy I is then used to remove buffer salts/enzymes and the large amounts of hydrolyzed glucose (typically 10–500x more abundant than the oxidation products). This comes with the cost of losing (new) reducing and oxidized termini as well. However, SPE produces solutions appropriate for UPLC-MS analysis, with the possibility to raise the analyte concentration by evaporation/reconstitution, while ensuring minimal interferences.

Figure 4 makes it clear that the major oxidized oligomer products detected after enzymatic digestion and SPE should in theory have two features in common, irrespective of the type of product:

- The β-(1→3)-linked reducing end unit, and
- The location of the oxidation-site at the non-reducing end.

**Figure 5:**
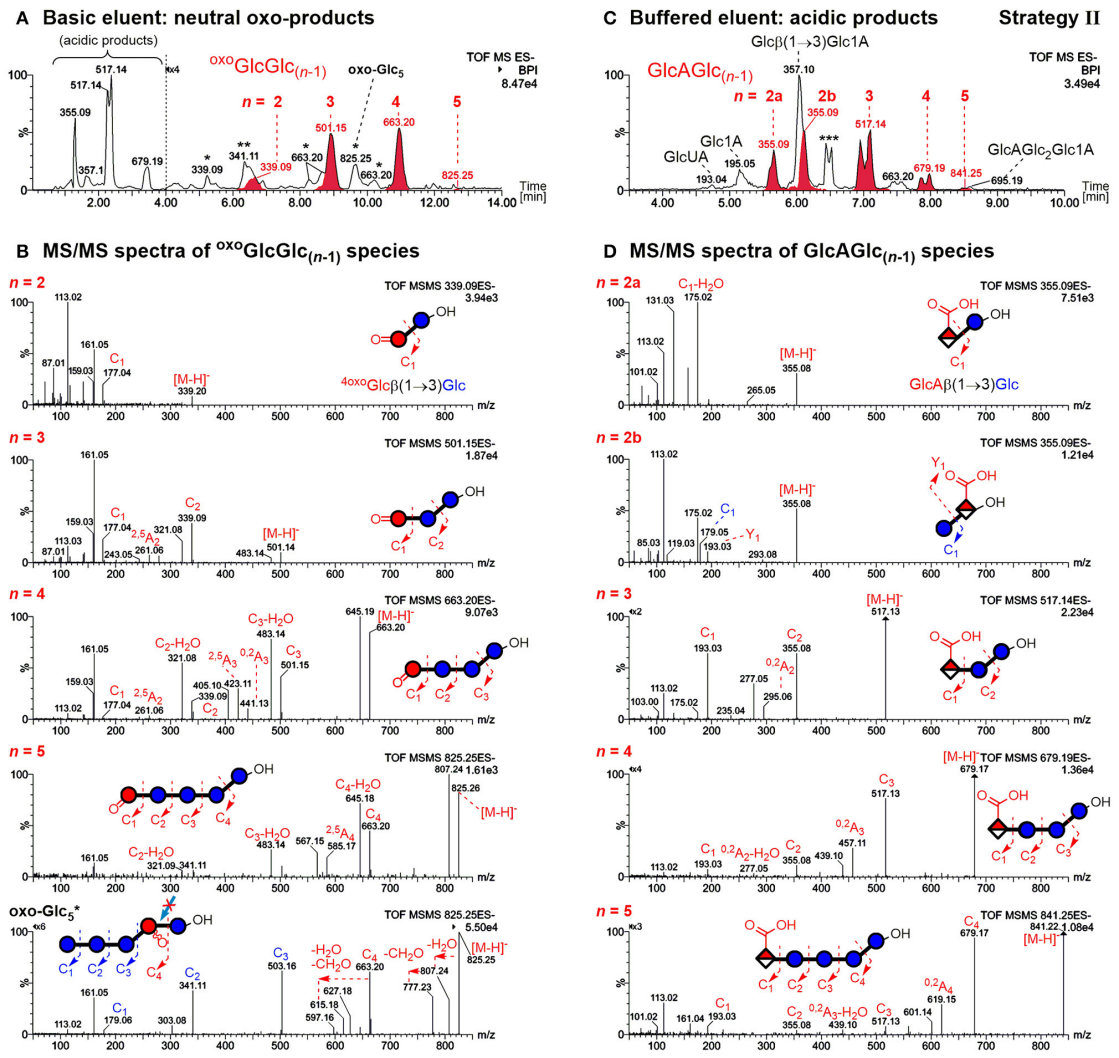
UPLC-MS/MS results in the negative mode using a BEH amide column and ACN/H_2_O gradient for analysis of barley β-glucan (BBG) oxidation products (harsh conditions) after enzymatic digestion and SPE (strategy II). **(A)** Analysis of the neutral products using basic 0.1% NH_3_ eluent additive (oxo-product peaks with carbonyl group at the non-reducing end ^oxo^GlcGlc_(*n*−1)_ in red) and **(B)** their respective MS/MS spectra. The main oxo-Glc_5_ species with C=O at a different position is also included (bottom spectrum). **(C)** Analysis of acidic products using a buffered 60 mM ammonium formate eluent additive (pH ~ 8) with glucuronic acid bearing oligomers GlcAGlc_(*n*−1)_ in red. **(D)** Respective MS/MS spectra of acidic products. The MS/MS fragments are named according to the nomenclature of Domon and Costello (1988) (cleaved linkage: C_*i*_ fragments; cross-ring cleavage: A_*i*_ fragments; *i* = 1, 2, … *n*). ^*^oxo-products with oxidized carbonyl group not at the non-reducing end; ^**^Disaccharide signal from catalase material; ^***^Buffer salt/solvent peak.

The former allows differentiation of mixed-linkage BG oxidation from e.g. cellulose, which only has β-(1→4)-linkages. The resulting UPLC-MS chromatograms of the prepared sample and MS/MS of the detected BBG products are shown in Figure 5 using basic (0.1% NH_3_) and buffered (60 mM NH_4_HCO_2_) eluent systems for the neutral oxo-Glc_*n*_ and the acidic products, respectively.

##### Acidic products

As previously reported, the acidic products are poorly retained on the BEH amide column with the basic eluent, allowing the differentiation of acidic vs. neutral products (Boulos and Nyström, 2016). The observed C6-oxidation products GlcAGlc_(*n*−1)_ with *n* = 2–5 could be separated according to size by UPLC with buffered eluent, having the expected (i) β-(1→3)-linked reducing end unit and (ii) C6-oxidation at the non-reducing end (see Figure 5C). This could be confirmed by negative mode MS/MS on the basis of fragmentation mechanisms proposed in our previous model oligo-BG oxidation study (nomenclature according to Domon and Costello, 1988). In short, fragmentation occurs largely unidirectional in negative mode from reducing end (C_*n*_ fragment) to non-reducing end (C_1_ fragment), and cross-ring cleavage fragments (e.g., ^0,2^A_*i*_ & ^2,5^A_*i*_; *i* = 1, 2,… *n*) are observed for β-(1→4)-, but not for β-(1→3)-linkages (Boulos and Nyström, 2016). Note that split peaks of *m*/*z* 517 (*n* = 3) and *m*/*z* 679 (*n* = 4) are due to anomers of the reducing end that do not coalesce under buffered eluent conditions.

**Figure 6:**
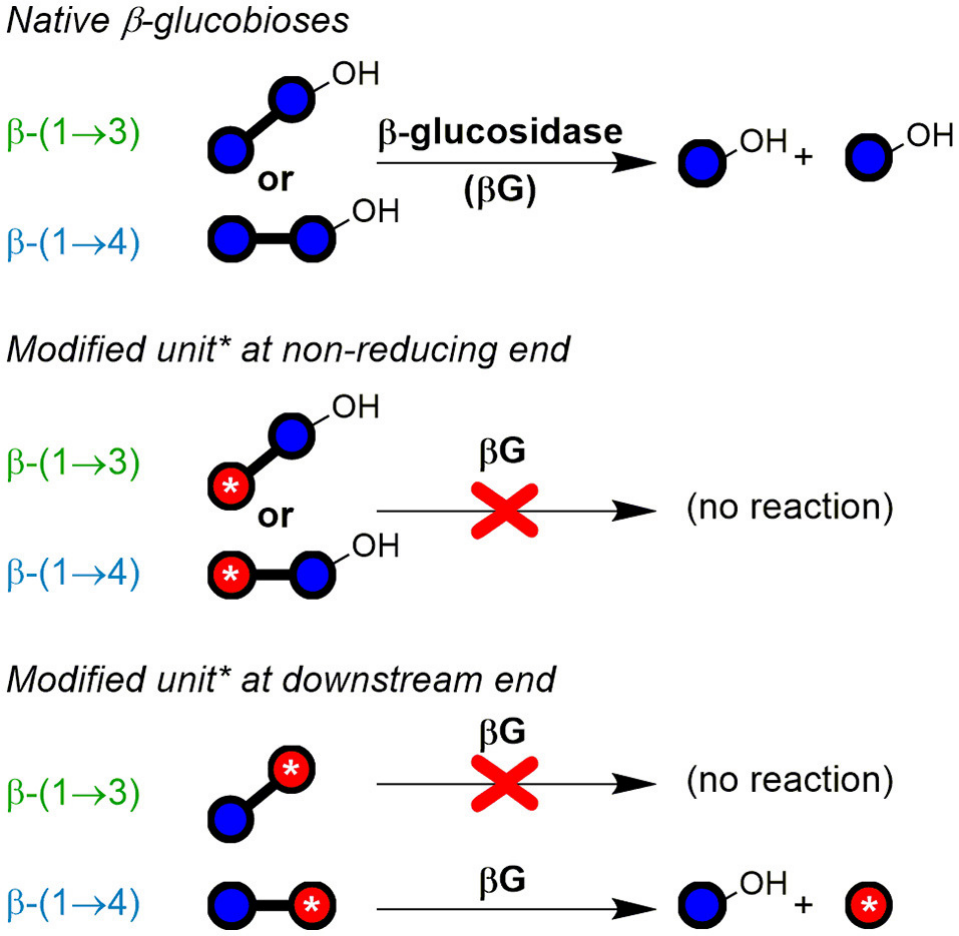
Susceptibility of native and modified β-glucobioses to hydrolysis by the used *exo*-β-glucosidase (from *Aspergillus niger*; EC 3.2.1.21; GH family 3). The enzyme was observed to cleave off native β-(1→3)- and β-(1→4)-linked non-reducing end glucosyl units, unless they are β-(1→3)-linked to certain types of modified units (red ○ with ^*^), especially if containing CO_2_H or labeled functional groups. Blue ○, glucose unit; blue ○–OH, reducing end glucose unit.

For the disaccharide GlcAGlc of *m*/*z* 355 ([M-H]^−^), however, two peaks could be observed. One is consistent with the expected GlcAβ(1→3)Glc product (*n* = 2a), whereas the other was assigned to Glcβ(1→3)GlcA (*n* = 2b in Figures 5C,D) by MS/MS. In addition, gluconic acid end groups from lytic C1-oxidation were also not fully hydrolyzed by β-glucosidase, as *m*/*z* 357 (eluting at ~6 min) could be identified as Glcβ(1→3)Glc1A by MS/MS and comparison with a prepared standard (Boulos and Nyström, 2016). In contrast, no β-(1→4)-linked disaccharides were detected. Apparently, some disaccharides with a modified glucose unit (Glc1A, GlcA) as the β-(1→3)-linked downstream (reducing) end unit are resistant to the used β-glucosidase from *Aspergillus niger* on the time scales used for the treatment (max. 1 day) despite the native non-reducing glucose unit, whereas the respective species with β-(1→4)-linkage seem to behave as expected and are fully hydrolyzed to the monomers (Figure 6). It is the combination of β-(1→3)-linkage plus modification of the 3-O-glucosylated unit that leads to resistance, and not the β-(1→3)-linkage alone, as native isomeric β-(1→3, 1→4)-gluco- and cello-oligomers are known to be equally good substrates for this β-glucosidase regardless of the β-(1→3)-linkage being located at the reducing end, the non-reducing end, or no β-(1→3)-linkage at all [only β-(1→4)] (McCleary and Harrington, 1988). Information on oxidized end units from C1-oxidation is thus partially conserved in the case of β-(1→3)-linkage (in theory ~30%) as the detected disaccharide, whereas information on β-(1→4)-linked Glc1A is mostly lost due to their observed complete hydrolysis and poor retention of the resulting Glc1A monosaccharide during SPE fractionation.

##### Non-reducing end oxo-products

Elution behavior of oxidation products with a carbonyl group at the non-reducing end ^oxo^GlcGlc_(*n*−1)_ (red peaks in Figure 5A) is in accordance with the made observations in an earlier study of BG oligomer oxidation, namely eluting later than their respective native oligomer (Boulos and Nyström, 2016). MS/MS confirms for all four oligomers (*n* = 2–5) of this type again the expected β-(1→3)-linked reducing end unit and location of the carbonyl group at the non-reducing end (Figure 5B). It cannot, however, determine the exact location of the carbonyl on the glucose unit. Those observed oxo-oligomers might thus be a mixture of products resulting from cleavage at C3/4, and from a non-lytic oxidation of a hydroxyl group (e.g., at C2 as shown in Figure 4). For the latter, the subsequent removal of native glucose units from the non-reducing end by β-glucosidase is responsible for the final location of the oxidized unit as the non-reducing end (Figure 4; final oligomer product on the bottom right), just like for lytic C4-oxidation (bottom left). However, from comparison of the relative amounts of the main species *m*/*z* 501 (*n* = 3) and *m*/*z* 663 (*n* = 4) before and after β-glucosidase (not shown), we suspect that most of the detected ^oxo^GlcGlc_(*n*−1)_ signals after β-glucosidase originate directly from lytic C3/C4 oxidation (colored in red in Figure 5A).

##### Mid-chain and reducing end oxo-products

MS/MS for the rest of the additional isobaric peaks for *n* = 2–5 eluting earlier than their ^oxo^GlcGlc_(*n*−1)_ counterpart (see Figure 5A, *m*/*z* 663 and 825 peaks labeled with ^*^) revealed the carbonyl to be located on a mid-chain unit or at the reducing end, making some of them non-lytic oxo-products (see Figure S3 for MS/MS). For *n* = 2 (*m*/*z* 339^*^ at ~5.3 min), one can imagine this oxo-product to be a Glcβ(1→3)^oxo^Glc species, e.g. from lytic C5-oxidation, consistent with being resistant to β-glucosidase due to the oxidized β-(1→3)-linked reducing end as was the case for GlcGlcA (Figure 6). For *n* = 5, the isobaric oxo-Glc_*n*_ peak even happens to be the main isomer (oxo-${\text{Glc}}_{5}^{*}$ peak at ~9.7 min). MS/MS of the main oxo-Glc_5_ product is consistent with the carbonyl group at a β-(1→3)-linked unit next to the reducing end, as it has the same type of fragmentation patterns (loss of H_2_O and CH_2_O) as determined to be diagnostic for cereal BG oxidation in our previous BG model compound study (Figure 5B, bottom MS/MS; Boulos and Nyström, 2016). Thereby, certain oxidation sites might hinder lichenase from hydrolyzing according to its general activity, explaining why products such as oxo-${\text{Glc}}_{5}^{*}$ were not cleaved to the oxo-Glc_4_ species (lichenase resistant site marked with blue arrow in proposed oxo-${\text{Glc}}_{5}^{*}$ structure in Figure 5B). Precedence for lichenase' inability to hydrolyze certain β-(1→4)-linkages despite their location next to a β-(1→3)-linked unit exists, namely if located at the non-reducing end (such as G_1_-^3^**G**_1_**-**^4^**G**_1_-^4^G_1_-^4^G_1_-^3^*G*, surprisingly resistant linkage in bold; Simmons et al., 2013). However, it is unclear why the non-reducing end portion of several native glucose units were not removed by β-glucosidase in substrates such as oxo-${\text{Glc}}_{5}^{*}$. This is subject to further investigation.

In addition, the mild oxidation (250 μM AH_2_) led to different proportions of oxo-Glc_*n*_ products, with ^oxo^GlcGlc_(*n*−1)_ (C=O at non-reducing end) not being the predominant product for *n* = 3, 4 as is the case under the harsh degradation conditions (100 mM H_2_O_2_; see Figure S4 for extracted ion chromatograms). We speculate that some oxo-Glc products are more susceptible to secondary oxidation than others. A likely candidate would be 6-oxo-glucose units containing species, whose C6 aldehyde group might easily oxidize to the respective C6 carboxylate to form glucuronic acid (GlcA) under the harsh conditions, which would explain the diminished number of isobaric oxo-Glc_*n*_ peaks compared with the mild oxidation.

Nonetheless, the unexpected resistance toward the enzyme of some oligomers with mid-chain & reducing end carbonyl location, namely oxo-Glc_*n*_ products labeled with ^*^ in Figure 5A, can be attributed mostly to non-lytic oxidation products. Hence, products from lytic C3/C4 action can be easily distinguished from the non-lytic action by the retention time and MS/MS of the detected oxo-Glc_*n*_ species after lichenase/β-glucosidase digestion and SPE. Additionally, ionization in the positive mode allowed differentiation of isobaric oxo-species due to different adduct formation preferences (e.g., [M+Na+H_2_O]^+^ for ^oxo^GlcGlc_(*n*−1)_ vs. [M+Na]^+^ for other oxo-Glc_*n*_) depending on C=O-location (see Figure S5).

#### C=O labeling strategy III: (new) reducing ends

**Figure 7:**
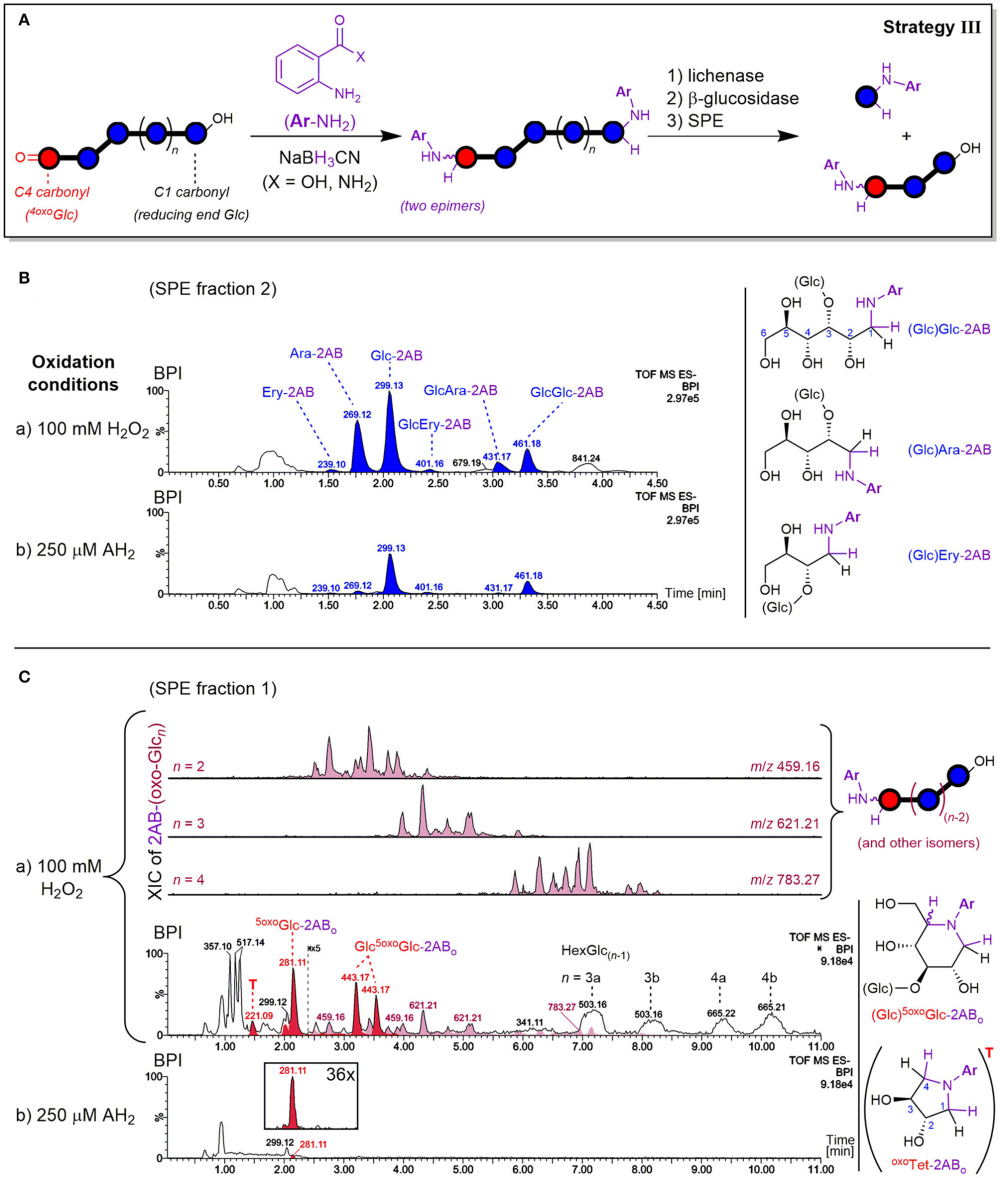
**(A)** Depiction of carbonyl labeling strategy III by reductive amination with anthranilic acid (2-AA; X = OH) or 2-aminobenzamide (2-AB; X = NH_2_) with enzyme digestion and SPE steps analogous to strategy II (see Figure 4). Negative mode UPLC-MS base peak chromatograms (BPI; basic eluent) resulting from oxidation of barley β-glucan (BBG) under a) harsh and b) mild conditions after the C=O sample preparation are shown for **(B)** SPE fraction 2 containing labeled reducing termini, and **(C)** SPE fraction 1 containing labeled oxo-products with extracted ion chromatograms (XIC) of 2AB-(oxo-Glc_*n*_). BPI of **(C)** were obtained after BPI-subtraction of the control sample (0.6% BBG) subjected to the same sample preparation, and the insert under b) shows the 36x zoomed XIC of *m*/*z* 281. AH_2_, ascorbic acid; Glc, glucose; Ara, arabinose; Ery, erythrose; Ar, aromatic ring; ^5oxo^Glc-2AB_o_, presumed cyclic product from 5-oxo-reducing ends (see Figure 8); ^oxo^Tet-2AB_o_ = “T,” presumed cyclic product of l-*threo*-tetrodialdose (*m*/*z* 221); 2AB-(oxo-Glc_*n*_), labeled oxo-Glc_*n*_ at oxidized C=O group; HexGlc_(*n*−1)_, oligosaccharide with one sugar unit being an undefined hexose (side products, presumably from direct reduction of oxo-Glc_*n*_; see Figure S9).

The principle of the carbonyl labeling strategy III is shown in Figure 7A. Reductive amination tags the carbonyls formed through oxidation as well as the reducing ends, which are naturally present but also newly formed during the degradation (e.g., by lytic C3/C4 oxidation, see Figure 2). This results in the same types of oligomer products after enzyme treatment as in strategy II. The main difference lies in the conserved information of the reducing ends that get lost without labeling, as they cannot be distinguished from glucose originating from non-oxidized parts of the polymer released by β-glucosidase if untagged. As labeled glucose, on the other hand, they are retained on SPE thanks to their bigger size, higher hydrophobicity, or added charge. Carbonyl labeling by reductive amination with NaBH_3_CN was conducted using two labels under different conditions, namely harsher conditions for labeling with 2-aminobenzamide (2-AB; 80°C with AcOH additive), and milder conditions for anthranilic acid (2-AA; 65°C). This allowed for the comparison of product profile proportions to validate the results and determine the occurrence of potential side reactions.

##### Detected reducing ends

Both amines 2-AB and 2-AA gave labeled products with neutral and charged character, respectively, owing to their different substitution on the phenyl ring (CONH_2_ vs. CO_2_H), leading to differing behavior in elution. As for the neutral and acidic products from the enzyme digested oxidized BG (strategy II), neutral 2-AB products were best analyzed with the basic 0.1% NH_3_ eluent, and acidic 2-AA products with the buffered eluent ideal for carboxylic acids (60 mM NH_4_HCO_2_, pH~8). Both labeling procedures ran to full conversion with no alditol side products, and gave the same type of products in essentially the same relative proportions. Thereby, SPE fraction 2 contained most products with labeled Glc and Glc_2_ being the most prominent signals, followed by cross-ring cleavage products Ara and GlcAra, and faint Ery and GlcEry signals (Figure 7B). These labeled neutral mono- and disaccharides represent actual carbonyls from reducing ends. As for the enzyme digestion products without labels, where Glcβ(1→3)Glc1A and Glcβ(1→3)GlcA turned out to be resistant to β-glucosidase (Figure 6), the labeled glucobiose was identified to be β-(1→3)-linked by comparison of MS/MS and retention time with labeled standards (see Figure S6).

The mild oxidation (250 μM AH_2_) gave similar signal strength of labeled reducing end Glc-2AB as the harsh oxidation (100 mM H_2_O_2_). The possibility of hydrolysis occurring during the reductive amination as an explanation for the similar Glc-2AB signals in the harsh and mild oxidation could be excluded, as the corresponding 2-AA species from the milder labeling-condition resulted in the same relative proportions for Glc-2AA from AH_2_ vs. H_2_O_2_. Additionally, control experiments with oligomers as substrate, where no smaller labeled oligomers and hence no hydrolysis could be detected under identical conditions (not shown). Interestingly, however, the two oxidation conditions exhibited different proportions regarding the more diagnostic cross-ring cleavage products such as Ara- and Ery-2AB (Figure 7B). This contrasts results from Mäkelä et al. (2017), who also observed arabinose from OBG and BBG oxidation with H_2_O_2_ (10–70 mM) by HPAEC-PAD after hydrolysis with lichenase/β-glucosidase, but not for samples oxidized with AH_2_ (10–70 mM). This underlines the sensitivity of our method with 2-AB labeling and UPLC-MS analysis that allowed detection of cross-ring cleavage products even from a very mild oxidation.

##### Lytic C5-oxidation

**Figure 8:**
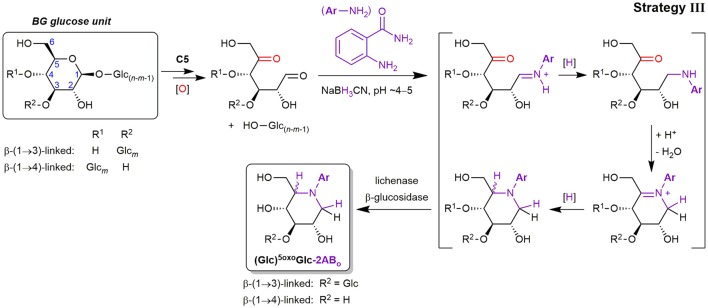
Lytic C5-oxidation forming a new terminus ^5oxo^Glc under loss of the non-reducing end-portion of the β-glucan polymer, and its reductive amination with 2-aminobenzamide (2-AB) (C=O labeling strategy III). The shown mechanism explains the cyclic end products ^5oxo^Glc-2AB_o_ and Glcβ(1→3)^5oxo^Glc-2AB_o_ detected in SPE fraction 1), which involves two reductive amination steps to form the piperidine derivative (first intermolecular tagging of the reducing end carbonyl (C1), then intramolecular with the C5-ketone). Glc, Glucose; [O], oxidation (Fenton-induced); [H], reduction (hydride from NaBH_3_CN).

Apart from the described products predominantly detected in SPE fraction 2, fraction 1 contained one major product with *m*/*z* 281.11 (in the case of 2-AB), which is smaller by 18 Da = H_2_O compared to Glc-2AB (*m*/*z* 299.13; see Figure 7C). Two peaks of *m*/*z* 443.17 were also found, which are 162 Da = one anhydroglucose unit larger than *m*/*z* 281.11. These products were also detected in fraction 1 with 2-AA as the label (*m*/*z* 282.10 and 444.15). The two species could be assigned to labeled ^5oxo^Glc and Glcβ(1→3)^5oxo^Glc, if instead of tagging the two carbonyls (reducing end (C1) and C5) with two amino-labels, only one label does the job for both positions, with epimers from the reduction explaining the double peaks for the disaccharide (for a rationalization of epimer proportions with regard to linkage type, see Figure S7). This is possible, as the spatial distance of carbonyls in this δ-keto-aldehyde allows for a unique mode of labeling by reductive amination: a second, intramolecular reductive amination step resulting in cyclization to form a piperidine derivative (see Figure 8). With intramolecular reactions being often faster than intermolecular ones, especially when forming 6-membered rings, no double-tagged species 2AB-^5oxo^Glc-2AB could be detected.

Precedence for such a double reductive amination exists for ^5oxo^Glc and other dicarbonyl sugars as reported by Baxter and Reitz (1994), who exploited the ring-formation for the synthesis of aza-sugar building blocks. Since no other constellation of carbonyls in primary oxidation products with 6 carbons are expected to result in such a cyclization, and dehydration side products with the same *m*/*z* could be excluded, these products in fraction 1 could be ascribed to lytic C5-oxidation (see Figures S8A,B for MS/MS). We also found a peak of *m*/*z* 221.09 that could be assigned to labeled l-*threo*-tetrodialdose (^oxo^Tet-2AB_o_ in Figure 7C), which was also observed by Schuchmann and Sonntag (1977) in their Glc irradiation study under O_2_. It is like ^5oxo^Glc a product induced by HO^•^-mediated H-atom abstraction on C5, but undergoing an alternative pathway with additional cleavage of the C4–C5 bond, resulting in the loss of 2 carbons (for the reaction scheme, see Figure 2). The successful detection of lytic C5-oxidation products is fortunate, as ^5oxo^Glc is a clear marker for BG oxidation, in contrast to reducing ends that are already present before oxidation.

Interestingly, lytic C5-oxidation has been largely overlooked as a pathway for direct cleavage of glycosidic linkages in HO^•^-mediated polysaccharide oxidation under formation of ^5oxo^Glc reducing ends (Faure et al., 2014; Iurlaro et al., 2014). This recent lack of reporting on C5-oxidation is especially puzzling in light of HO^•^-attack being somewhat favored at the C5-position as determined by EPR studies (Park et al., 1999) and pulsed γ-radiolysis of cellobiose (Sonntag et al., 1976). Lindsay and Fry (2007) proposed for the intermediate C5-oxonium ion (R_2_C=O^+^R′; formed after HO^•^-attack at C5 and O_2_/${\text{O}}_{2}^{•-}$ addition/elimination), to undergo a tautomerisation that leads to labile Glc1A esters, instead of simply liberating ^5oxo^Glc through hydrolysis. However, such a direct, thermal [1,3]-hydride shift to Glc1A-esters is mechanistically not possible (Woodward and Hoffmann, 1969). We, on the other hand, have first hypothesized the occurrence of ^5oxo^Glc species as the direct result of lytic C5-oxidation under Fenton-conditions in our BG model compound oxidation study (Boulos and Nyström, 2016), but were also not able to unambiguously identify it by direct-injection UPLC-MS/MS due to complex isobaric product mixtures and sensitivity issues. In this study, however, with the oxidation of polymeric BG, C=O labeling, and enzymatic treatments that largely eliminate interferences and simplify product profiles by focusing only on carbonyls, we present for the first time evidence for a direct lytic C5-oxidation in BG under formation of 5-oxo-reducing ends.

##### Non-reducing end and mid-chain oxo-products

The other expected type of products is labeled ^oxo^GlcGlc_(*n*−1)_ or oxo-Glc_*n*_ in general. They could be found in SPE fraction 1 as complex mixtures [see extracted ion chromatograms (XIC) of *m*/*z* 459, 621, and 783 in Figure 7C]. As for the labeled cyclic ^5oxo^Glc, each oxo-product should lead to two epimers after the reductive amination due to the intermediate imin that can be reduced from two different faces (with the exception of C6-aldehyde, which leads to only one product). Thereby, each C=O location is expected to lead to different proportions of those epimers due to the respective steric situations (1:1 to up to 95% selectivity; Baxter and Reitz, 1994). Naturally, this complicates the product mixtures, which explains the observed higher number of peaks for these labeled oxo-Glc_*n*_ products than the respective non-labeled species from strategy II (Figure 5A). While the complexity of these isomeric product mixtures was expected, MS/MS did not suffice to unambiguously assign the peaks to specific products due to their similar fragmentation spectra (see Figures S8C,D for XIC and average MS/MS spectra).

Next to the acidic products also observed as in strategy II (e.g., *m*/*z* 357, 517) and the mentioned labeled oxo-species, broad peaks with masses isobaric to native glucosyl-oligomers were detected in SPE fraction 1 (*m*/*z* 503, 665; see Figure 7C). They are much more prominent than the labeled oxo-Glc_*n*_ products discussed above. It is possible that these oxo-products are partially lost by a side reaction of the reductive amination, forming the prominent unlabeled oligomers. The more sterically hindered keto-groups (compared to the reducing end aldehyde/hemiacetal) could slow down the initial imin-formation step, making a competing direct reduction to glucosyl epimers with NaBH_3_CN possible, which is known to reduce carbonyls at pH 3–4 as well (Lane, 1975). Detection of signals isobaric to Glc_*n*_ in elevated levels compared to the control, but with different retention times than the native BG oligomers from lichenase digestion, speaks for this hypothesis (see Figure S9).

Recently, Frommhagen et al. (2017) attempted to circumvent such a side reaction when labeling 4-oxo-Glc bearing oligomers produced from LPMO oxidation of cellulose by using NaBH(OAc)_3_ as reducing agent. However, they also observed direct reduction of the carbonyl. As an alternative, the authors investigated the direct non-reductive labeling to form the Schiff base imin selectively at the reducing end. However, the low degree of conversion to the imin and the reversibility of the labeling reaction impeded an effective quantification. Other solutions to optimize the labeling of non-reducing end/ mid-chain oxo-products are part of future studies. Nevertheless, the described method is suitable for labeling of the reducing end (including ^5oxo^Glc) with full conversion, conserving the information of neutral termini in BG oxidation.

#### CO_2_H labeling strategy IV: lytic and non-lytic acidic products

**Figure 9:**
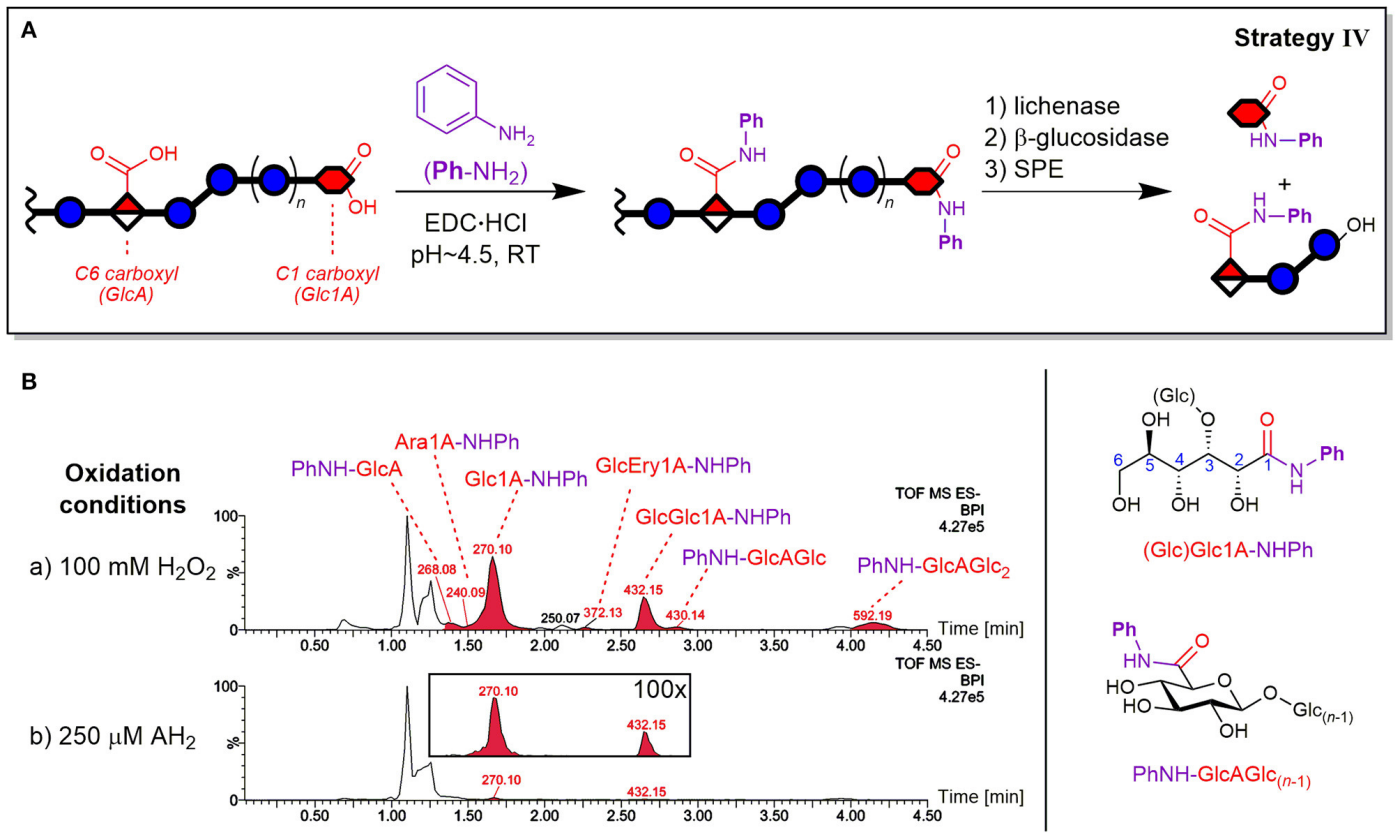
Depiction of **(A)** carboxylic acid labeling strategy IV by EDC-mediated amidation of oxidized β-glucan with aniline (PhNH_2_) and enzyme digestion/SPE steps analogous to strategy II (see Figure 4). **(B)** The resulting base peak ion chromatograms (BPI) from oxidation of barley β-glucan (BBG) under (a) harsh and (b) mild conditions after the CO_2_H sample preparation (basic eluent). The peaks are labeled with their base peak ion *m*/*z* and corresponding structure (chemical structures on the right-hand side). The insert under (b) shows 100x zoom of overlaid extracted ion chromatograms of *m*/*z* 270 and 432. Glc, glucose; Glc1A, gluconic acid; GlcA, glucuronic acid; Ara1A, arabinonic acid; Ery1A, erythronic acid; AH_2_, ascorbic acid; Ph, phenyl; -NHPh or PhNH-, aniline amide (anilide) of acid species; EDC, ethyl-3-(3-dimethylaminopropyl)carbodiimide.

Carboxylic acid labeling was accomplished by amidation *via* carbodiimide activation of CO_2_H using aniline (PhNH_2_) both as label and as pH buffer and was based on a procedure by Yang et al. (2008) for energy metabolism analysis (see Figure 9A).

##### Detected products as anilides

The expected oxidized gluconic acid end units from lytic C1 oxidation of BG degradation was the major product using the CO_2_H labeling sample preparation strategy IV (see Figure 9B). They were detected as Glc1A-anilides (Glc1A-NHPh, amide of aniline) after enzyme treatment/SPE, conserving information about lytic C1-oxidation that is otherwise (partially) lost in the enzymatic treatment without carboxylate tagging (strategy II), as the necessary SPE step only retains monosaccharides if labeled. In addition, gluconic acid was also detected as Glcβ(1→3)Glc1A-NHPh amide, apparently also resistant to β-glucosidase as the free acid GlcGlc1A itself (see Figure S10 for MS/MS spectra). Other oxidized termini that were conserved through labeling were the cross-ring cleavage products, which were observed in minor amounts as arabinonic and erythronic amides (Glc)Ara1A- and GlcEry1A-NHPh, respectively. Interestingly, the peak of Ara1A-NHPh was much smaller compared to Glc1A-NHPh (Figure 9B) than the respective peak of C=O labeled Ara-2AB compared to Glc-2AB termini (strategy III; Figure 7B). In addition, glucuronic acid products PhNH-GlcAGlc_(*n*−1)_ with *n* = 1–3 were also detected, representing non-lytic C6 oxidation. However, in contrast to strategy II, the labeled uronic acid species with *n* > 3 were only detected in traces, possibly due to the labeled GlcA unit rendering some linkages in their proximity not susceptible to lichenase anymore, resulting in larger oligomers (*n* > 8) that are not eluted from SPE and/or the UPLC-column under the chosen conditions. Hence, strategy IV is most useful for retaining the otherwise lost information on acidic termini.

##### CO_2_H-labeling efficiency

The reaction of carboxylates with PhNH_2_ did not lead to complete tagging, which was also observed for test reactions with (Glc)Glc1A and GlcA standards (not shown). This seems to be intrinsic to carbodiimide activated carboxylic acids in aqueous polysaccharide solutions, with, for example, typical degrees of substitutions of 0.1–0.3 for alginates (Cumpstey, 2013). A reason might lie in lactone formation after activation of the carboxylate instead of reaction with the amine (e.g., Glc1A + EDC→→ Glc1A-δ-lactone). Those lactones are not readily hydrolyzed back to the carboxylate at pH 4.5, and apparently do not (fully) open up with PhNH_2_ to form the amide. However, known side reactions such as rearrangement of EDC-activated carboxylates to stable *N*-acyl ureas (Valeur and Bradley, 2009) were not observed. Attempts for optimization, e.g., by using a different amine such as 2-AB, addition of *N*-hydroxysuccinimide (NHS), changing pH, testing alternative coupling reagents (e.g., PyBOP) and anhydrous conditions (in DMF) were not successful and lead to lower yields in test reactions with acid standards (not shown). Nevertheless, under our reaction conditions, the labeling of carboxylates in BG proved to be robust with constant conversions, therefore allowing comparison of different samples.

#### Comparison, scope, and limitations of the four strategies

The presented four complementary sample preparation strategies (I–IV) used to access different information about BG oxidation are summarized in Table 1, revealing similarities and differences of the various approaches (see Figure S11 for the extended version with product structures, symbols, names, and abbreviations; and Table S1 for lost vs. preserved information). The comparison makes it clear that SPE strategy I provides limited information and low sensitivity since it is confined to a narrow window of product size (*n* = 2–8; <1% of total BG material). The additional enzyme digestion prior to SPE (strategy II) leads to distinct types of products due to the removal of non-oxidized parts of the polymer in a well-defined fashion. This unlocks access to the information on the polymer and makes the method specific for detection of BG oxidation due to the specificity of lichenase for β-(1→3, 1→4)-mixed linkage glucans (Table 1). It also makes the method more sensitive, as most of the non-oxidized glucose units are removed, allowing for detection of prominent oxidation products also under the mild conditions with AH_2_ [oxo-Glc_*n*_ & GlcAGlc_(*n*−1)_].

**Table 1:** Comparison of four sample preparations with regard to UPLC-MS detectable β-glucan products of each method and their characteristics^a^.

**Type of detectable products/ method characteristics**	**Sample preparation strategy**			
	**I: SPE**	**II: Enzymes**	**III: C=O labeling**	**IV: CO_2_H labeling**
Polymeric products	No	Yes	Yes	Yes
Oligomeric products	Yes	Yes	Partially^d^	Partially^d^
Neutral (new) reducing ends	Partially^b^	No	Yes	No
^oxo^GlcGlc_(*n*−1)_ (C3/C4, lytic)	Partially^b^	Yes	Partially	No
^5oxo^Glc products (C5, lytic)	Partially^b^	Partially^c^	Yes	No
Non-lytic oxo-products	Partially^b^	Yes	Partially	No
Glc1A products (C1, lytic)	Partially^b^	Partially^c^	No	Yes
GlcA products (C6)	Partially^b^	Yes	No	Yes
Acidic cross-ring cleavage	Partially^b^	Partially^c^	No	Yes
Sensitivity (regarding mild oxidation)	Low	Medium	High	High
Specificity for cereal BG	Low	High	Medium	Medium
UV/fluorescent detection	No	No	Yes	Yes

a *See section Materials and Methods for details on the sample preparation strategies I–IV. Detectable products in the case of the two labeling procedures refer to only labeled products. Glc, glucose; Glc1A, gluconic acid; GlcA, glucuronic acid; ^5oxo^Glc, 5-keto-glucose; ^oxo^GlcGlc_(*n*−1)_, oxidized gluco-oligomer with new carbonyl at non-reducing end*.

b *Only oligomeric forms detected (information on polymeric products lost). Mixtures of isomers*.

c *Only β-(1→3)-linked oxidized units detected as disaccharides (theoretically ~30% of those termini)*.

d *Partial loss of small oligomeric products possible due to precipitation and washing procedure after labeling*.

Information on certain termini and their formation, namely reducing ends (Glc, Ara, Ery), lytic C1- and C5-oxidation (Glc1A & ^5oxo^Glc), though, are mostly lost due to hydrolysis to monomers and their lack of retention on SPE. Strategies III and IV take care of these neutral and acidic termini by C=O and CO_2_H labeling, respectively, while increasing sensitivity and making in principle an additional mode of detection possible, namely by UV/fluorescence. This has potential as it would allow in the future the quantification of certain products by taking advantage of the equal response factor regardless of tagged molecule. Due to side reactions for C=O labeling strategy III and presumably incomplete hydrolysis of CO_2_H labeled species by lichenase in strategy IV, strategy II is still best suited to assess lytic C3/C4 and non-lytic C6 oxidation [*via* ^oxo^GlcGlc_(*n*−1)_ & GlcAGlc_(*n*−1)_, respectively]. However, due to the extensive washing/precipitation steps, none of the sample preparation strategies in their current form capture small oxidation fragments that are not covalently bound to the polymer (C_*n*_ aldehydes/acids with number of carbons *n* < 6). A prominent example is formic acid (C_1_) which is released as co-product during the cross-ring cleavage to produce e.g., Ara and Ery termini (see Figure 2; Boulos and Nyström, 2016; Mäkelä et al., 2017). While these free C_1_, C_2_, C_3_ … acids technically constitute β-glucan oxidation products, they are not part of the oxidized polymeric β-glucan material that is of interest with regard to its altered physicochemical properties. In addition, the Ara/Ery & Ara1A/Ery1A termini detected by C=O- and CO_2_H-labeling strategies III and IV, respectively, implicitly carry the information on the amount of free C_*n*<6_ acids or aldehydes. To summarize, combining the complementary sample preparation strategies II, III, and IV provides a complete picture of BG oxidation with regard to relevant oligomeric and polymeric products, with some overlapping areas of detected product types serving as confirmation and validation of the method.

Regarding diagnostic value and sensitivity for detection of BG oxidation by UPLC-MS, the oxidized species obtained from labeling strategies III (C=O) and IV (CO_2_H) are the most promising candidates. We present for the first time in the Fenton-induced degradation of BG evidence for a direct lytic C5-oxidation under formation of 5-oxo-reducing ends—a process largely overlooked in the literature. With sample preparation strategy III, ^5oxo^Glc, and Glcβ(1→3)^5oxo^Glc were successfully detected in substantial amounts by UPLC-MS as labeled heterocyclic amines resulting from cyclization by an intramolecular reductive amination step unique to δ-keto-aldehydes. In contrast to reducing end units, which are already present before oxidation and can in real life samples also be produced by hydrolytic processes catalyzed by acids or enzymes, 5-oxoglucose is a clear marker for BG oxidation. It shows next to labeled cross-ring cleavage products arabinose and erythrose the highest potential for future use in determining extent of oxidation during food processing and storage.

### Part 2—application

#### Co-oxidation of BG and starch

**Figure 10:**
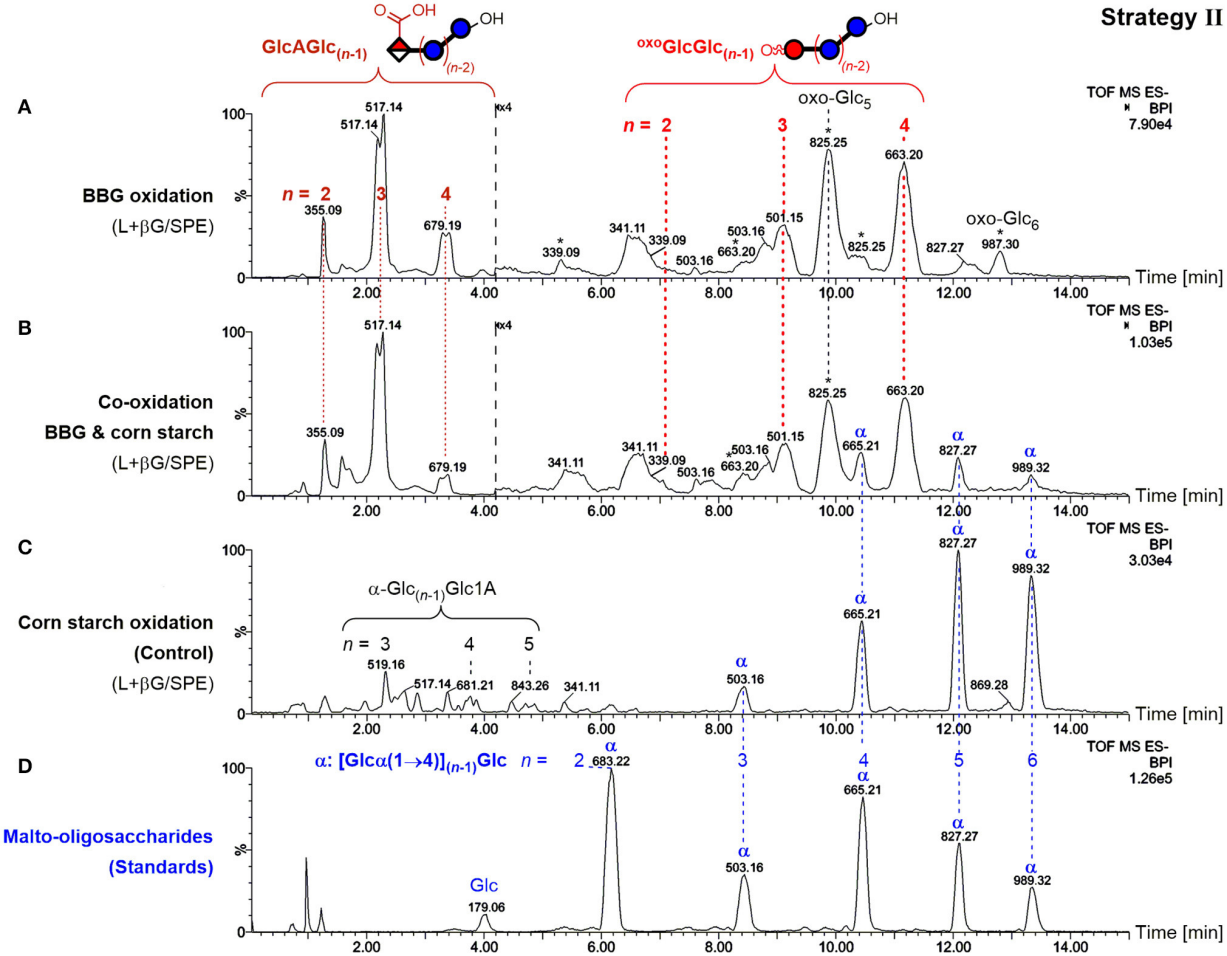
Comparison of UPLC-MS base-peak ion (BPI) chromatograms of polysaccharide oxidation under harsh conditions (100 mM H_2_O_2_, 50 μM FeSO_4_) after lichenase and β-glucosidase treatment/SPE for **(A)** only barley β-glucan (BBG) (0.6%; as in Figure 5A), **(B)** BBG and corn starch (each 0.6%), and **(C)** only corn starch (0.6%). **(D)** UPLC-MS of malto-oligosaccharide standards up to maltohexaose (*n* = 6). Glc, glucose; Glc1A, gluconic acid; GlcA, glucuronic acid; ^oxo^Glc, oxo-glucose; oxo-Glc_*n*_, gluco-oligomer with oxidized carbonyl group anywhere along the chain; ^oxo^GlcGlc_(*n*−1)_, gluco-oligomer with oxidized carbonyl at non-reducing end; L+βG/SPE, after lichenase and β-glucosidase treatment, followed by fractionation by SPE; ^*^, oxo-Glc_*n*_ with oxidized C=O not at the non-reducing end; α, malto-oligosaccharides [Glc_*n*_ with all α-(1→4)].

To showcase the specificity of the presented strategies, BBG was oxidized in the presence of cornstarch in equal amounts and subjected to sample preparation II (enzyme digestion/SPE) as an example. The use of enzymes specific for mixed-linkage BG (lichenase) leads to the exclusive hydrolysis of BG to form small oxidized oligomers resistant to β-glucosidase. Oxidized amylose/amylopectin from starch, on the other hand, should remain predominantly in their large, polymeric forms, and only a minor portion small enough to be retained in the subsequent SPE step should be observed (as in strategy I). Separation by the UPLC BEH amide column, which can resolve malto- from cello- and β-(1→3,1→4) mixed-linkage oligomers, in combination with MS/MS should undoubtedly distinguish any starch-oligomers from the BG oxidation products through the latter's β-(1→3)-linkage at the reducing end, and the expected oxidation site at the non-reducing end for the main GlcA- and oxo-products (Maina et al., 2013; Boulos and Nyström, 2016). And indeed, the BBG oxidation products with expected *m*/*z*, retention time, and MS/MS pattern could be detected in the co-oxidation (Figure 10B), with little difference on the profile compared to the oxidation without starch (Figure 10A as in Figure 5A). Some additional peaks were observed that could be assigned to native malto-oligosaccharides α-Glc_*n*_ and their C1 oxidized α-Glc_(*n*−1)_Glc1A (Figure 10C), representing different product types compared to BBG's GlcAGlc_(*n*−1)_ and ^oxo^GlcGlc_(*n*−1)_/oxo-Glc_*n*_ altogether, allowing together with MS/MS and comparison with standards (Figure 10D) their unambiguous differentiation.

This serves as a proof of concept for the specific detection of BG oxidation in the presence of other polysaccharides, making it clear that this sample preparation strategy II (and with minor method adjustments III and IV as well since lichenase is involved) should in principle be able to distinguish BG oxidation from oxidation of any other polysaccharide relevant to food systems (e.g., starch, cellulose, arabinoxylan, inulin, pectin, agarose, galactomannan…), provided they are not cleavable by lichenase, which—short of lichenin in Icelandic moss (Barras et al., 1969) and a mixed-linkage BG in certain *Equisetum* (horsetail) species (Fry et al., 2008)—should pose no limitations.

#### Relationship of BG fine structure DP3/DP4 and linkage type ratios of oxidation products

We analyzed how the fine structure differences of OBG vs. BBG, namely the ratios of β-(1→4)- to β-(1→3)-bonds and the cellotriosyl- to cellotetraosyl ratios of the polymer (equal to DP3/DP4), translate to the relative amounts of characteristic oligomeric oxidation products detected by the sample preparation strategies II-IV (see Table 2; results from harsh oxidation with 100 mM H_2_O_2_). The molar DP3/DP4 ratios of the starting materials were 1.7 and 2.7 for oat and barley, respectively, as determined by UPLC-MS after lichenase treatment of the native BGs and comparison with standards, and laid in the expected ranges (Lazaridou and Biliaderis, 2007). We also determined the ratios for the major ^oxo^GlcGlc_(*n*−1)_ and GlcAGlc_(*n*−1)_ species with *n* = 3 and 4 from enzyme digestion/SPE (strategy II).

**Table 2:** OBG vs. BBG comparison of DP3/DP4 ratios (obtained after lichenase treatment of the starting material) and the linkage type ratio, as well as their oxidized counterparts detected by UPLC-MS after lichenase and β-glucosidase digestion (strategy II) or functional group labeling (III and IV)^a^.

**BG source**	**DP3/DP4**	**^oxo^GlcGlc_(**n**−1)_ (*n* = 3)/(*n* = 4)**	**GlcAGlc_(**n**−1)_ (*n* = 3)/(*n* = 4)**	**β-(1→4)/β-(1→3)^b^**	**Glc*_*n*_*-2AA/AB (*n* = 1)/(*n* = 2)^c^**	**Glc_(**n**−1)_Glc1A-NHPh (*n* = 1)/(*n* = 2)^d^**
Oat	1.65	1.78	2.00	2.44	3.19	2.74
	(0.12)	(0.19)	(0.31)	(0.18)	(0.44)	(0.05)
Barley	2.71	1.73	2.91	2.33	2.99	2.65
	(0.12)	(0.10)	(0.06)	(0.10)	(0.41)	(0.16)
ratio Oat/Barley	0.65	1.02	0.69	1.05	1.07	1.04
	(0.05)	(0.11)	(0.11)	(0.09)	(0.15)	(0.06)
Oxidative process (carbon position)		Lytic C3/C4	Non-lytic C6	–	Lytic C3/C4	Lytic C1

a *Mean of MS peak area ratios from harsh oxidations (100 mM H_2_O_2_). Values in parenthesis refer to the standard deviations. Only DP3/DP4 refers to molar ratios, the rest refers to signal ratios. Glc, glucose; Glc1A, gluconic acid; GlcA, glucuronic acid; ^oxo^GlcGlc_(*n*−1)_, oxidized gluco-oligomer with new carbonyl at non-reducing end; GlcAGlc_(*n*−1)_, oligomer with GlcA at non-reducing end; Glc_*n*_-2AA/AB, aminodeoxyglucitol species from reducing end C=O-labeling by reductive amination with anthranilic acid (2-AA) or 2-aminobenzamide (2-AB) labels and NaBH_3_CN; Glc_(*n*−1)_Glc1A-NHPh, CO_2_H-labeled gluconic acid species as amides of aniline (PhNH_2_)*.

b *Linkage type ratio calculated from determined DP3/DP4 ratio of the native BG materials ignoring DP ≥ 5 (<10%)*.

c *Average of 2-AA & 2-AB-labeled Glc reducing end results after enzyme digestion/SPE (strategy III). The disaccharide refers to Glcβ(1→3)Glc-2AA (or 2AB) resistant to β-glucosidase*.

d *The disaccharide refers to Glcβ(1→3)Glc1A-NHPh resistant to β-glucosidase (strategy IV)*.

Since no standards are commercially available, the respective ratios in Table 2 refer to signal and not molar ratios, which does not give absolute values but still allows comparing the ratios with each other (Table 2, oat/barley). The results were mixed, with ^oxo^GlcGlc_(*n*−1)_ species having (*n* = 3)/(*n* = 4) ratios for OBG and BBG that were equal, whereas the GlcAGlc_(*n*−1)_ ratios with 2.0 and 2.9, respectively, differed depending on BG source. Analyzing the possibilities of formation systematically for these two types of products revealed the reason behind their different product ratio pattern.

##### ^oxo^GlcGlc_(n-1)_ ratios

**Figure 11:**
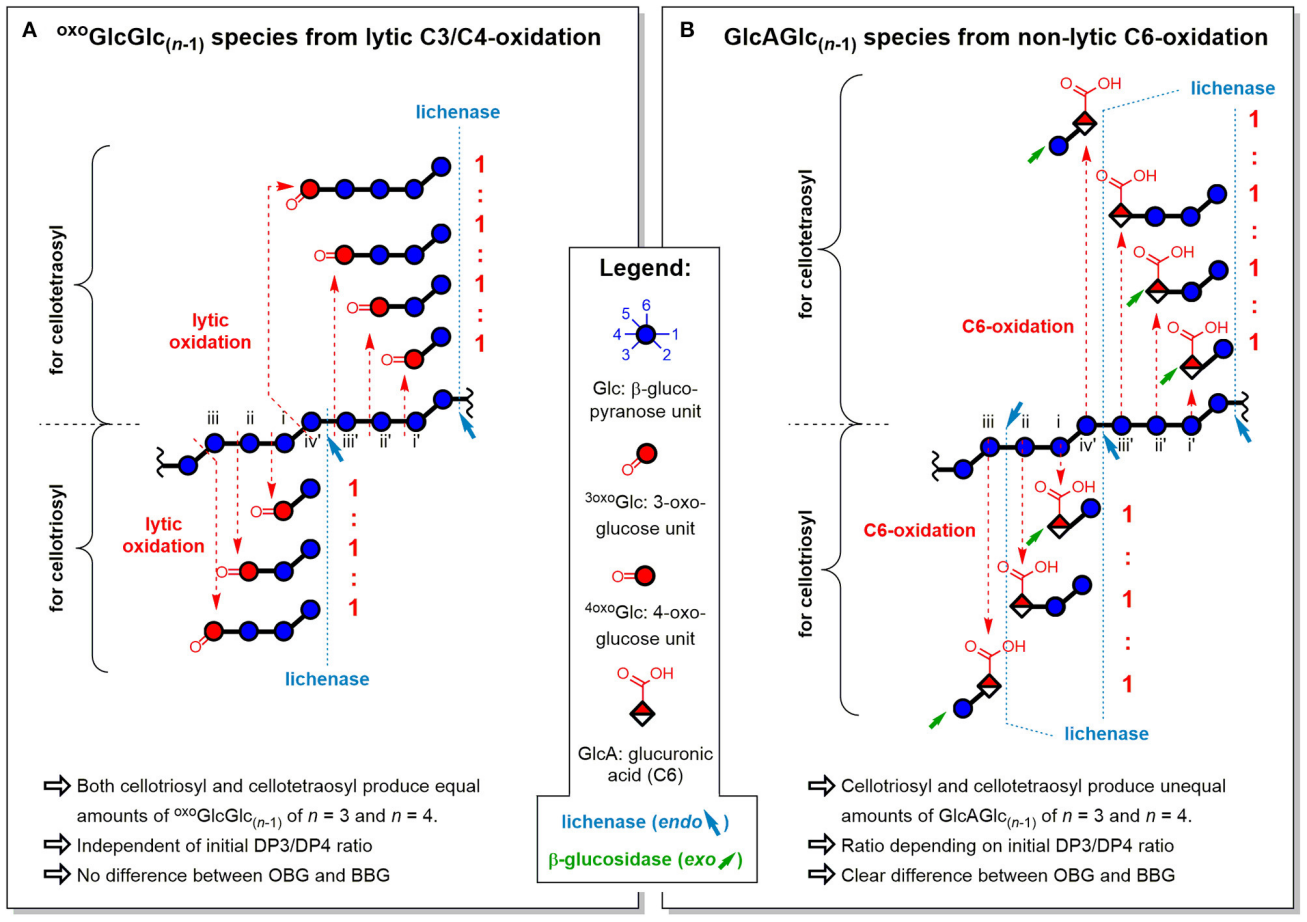
Influence of fine structure differences (DP3/DP4) of oat and barley β-glucan on the ratios of oligomeric oxidation products. Explanation for the resulting oligomeric oxidation product ratios (*n* = 3)/(*n* = 4) from **(A)** lytic C3/C4-oxidation and **(B)** non-lytic C6-oxidation, both after lichenase and β-glucosidase treatment/SPE (strategy II). ^oxo^GlcGlc_(*n*−1)_, gluco-oligomer with oxidized carbonyl at non-reducing end; GlcAGlc_(*n*−1)_, oligomer with glucuronic acid (C6-oxidized) unit at non-reducing end (unless for *n* = 2, where also at reducing end); BBG, barley β-glucan; OBG, oat β-glucan; DP3/DP4, molar ratio of cellotriosyl to cellotetraosyl units in β-glucan (defines fine structure).

The oxo-products originate mainly from lytic C3/C4 cleavage, meaning they can lose glucose units that are part of the cellotriosyl/cellotetraosyl fine structure, such that a resulting ^oxo^GlcGlc_(*n*−1)_ species with *n* = 3 not necessarily originates from a cellotriosyl substructure, but also from DP ≥ 4. Assuming equal probability of oxidative cleavage for each glycosidic linkage regardless of position, a cellotriosyl subunit can be oxidized to give—after lichenase and β-glucosidase—^oxo^GlcGlc_(*n*−1)_ with *n* = 2, 3, and 4 in a 1:1:1 ratio, whereas oxidation of a cellotetraosyl unit leads to oxo-products with *n* = 2, 3, 4, and 5 in a 1:1:1:1 ratio (see Figure 11A). The original DP3/DP4 ratio is thus not reflected in those lytic oxo-products, as both cellotriosyl and cellotetraosyl subunits can produce both oxo-Glc_3_ and oxo-Glc_4_ equally, explaining the identical ratios for OBG and BBG.

##### GlcAGlc_(n-1)_ ratios

The ratios of glucuronic acid species, on the other hand, are according to the native DP3/DP4 ratio (BBG>OBG; Table 2). Since introduction of a carboxylic acid at C6 is a non-lytic process, the possibilities of formation are different from the lytic oxo-products above. Here, a cellotriosyl subunit can lead to GlcAGlc_(*n*−1)_ with *n* = 2a, 2b, and 3 after enzymatic treatment, while for cellotetraosyl, glucuronic acids with *n* = 2a, 2b, 3, and 4 can be formed [2a and 2b species refer to β-(1→3)-linked GlcAGlc and GlcGlcA, respectively; see Figures 5C,D]. Hence, GlcAGlc_(*n*−1)_ with *n* = 4 cannot be formed from cellotriosyl units, whereas the *n* = 3 species can be formed from both cellotriosyl and cellotetraosyl units, making the C6-oxidation products depend on the DP3/DP4 ratio of the original starting material (see Figure 11B).

##### β-(1→4)/β-(1→3) vs. monosaccharide/disaccharide termini

As for the linkage type ratios, the resistance toward β-glucosidase hydrolysis of β-(1→3)-linked disaccharide products with labeled end units is used as an advantage for additional information on the randomness of the degradation. Thereby, aldonic acid CO_2_H labeling provides information about the selectivity on the lytic C1-oxidation process, and reducing end C=O labeling about the lytic C3/C4-oxidation process of the previously neighboring sugar unit (primary mode for releasing new reducing ends; Table 2, bottom row). The ratio of labeled mono- to disaccharide should directly reflect the β-(1→4)/β-(1→3) linkage ratio of the BG material, unless one linkage type is preferably cleaved, skewing the ratio toward one side or the other. The observed ratios of labeled mono- to disaccharide are 7 and 4% higher for OBG in the C=O- and CO_2_H-labeling, respectively, and in good agreement with the calculated ~5% higher β-(1→4)/β-(1→3) ratios for the used BG materials from oat vs. barley (see Table 2).

##### Implications on HO^•^-attack preferences

As discussed above, no significant differences could be found between the calculated theoretical DP3/DP4 ratios (based on the assumption of equal attack) and the experimental results regarding the lytic C3/C4- and non-lytic C6-oxidation oligo-products, as well as the linkage-ratio reflected in Glc1A and Glc reducing termini (labeled mono- vs. disaccharides). This implies equal probability of attack on each unit along the mixed-linkage chain for lytic as well as non-lytic processes within the accuracy of the measurements. Yet, estimated minor differences of ≤ 10% in preferential attack depending on the repeating unit's neighborhood cannot be excluded, as they might be masked by the experimental uncertainty and the averaging nature of some of these ratios. Nevertheless, the results corroborate earlier findings from a study using constitutionally isomeric BG model compounds (Glc_4_) with and without β-(1→3)-linkage, exhibiting no significant differences in their half-lives for the oxidative degradation (Boulos and Nyström, 2016). This is contrary to previous claims of preferred β-(1→3)-cleavage in a BBG oxidation study, where the linkage types were assessed by ^13^C-NMR analysis during the degradation (Faure et al., 2014). However, the degree of oxidation was much higher in the NMR study as evident by the *M*_*w*_ of BG being too low to be detectable by dynamic light scattering already after 2 h (85°C, 100 mM H_2_O_2_). The reported faster disappearance of the C3-glycosidic linkage signal could therefore be the result of late stages in the degradation that are rarely reached under physiological or food processing conditions. Hence, we conclude that the kinetic differences in the cleavage of β-(1→3)- vs. β-(1→4)-glycosidic bonds induced by the Fenton reaction average out to be either non-existent or too small to have any significant effect on the formation of products from lytic oxidation at C1 or C3/C4.

#### Relationship of BG molar mass with content of termini from lytic oxidations

The number average molar masses (*M*_*n*_) of the OBG and BBG starting materials as well as their values after 24 h oxidation under the two oxidation conditions (a) 100 mM H_2_O_2_ and (b) 250 μM AH_2_ were determined by SEC. They are listed in Table 3, showing that the harsher H_2_O_2_ conditions led to a ~20x reduction of *M*_*n*_, about 8x more drastic regarding loss in polymer length than the mild AH_2_ conditions. Using the molar masses, the theoretical reducing end contents can be calculated for each reaction (proportional to 1/*M*_*n*_, assuming all ends are in the form of Glc), which were set relative to the reducing end group (REG) content of the mild condition (AH_2_). This can then be compared with the relative UPLC-MS signals of termini detected with enzymes/SPE strategy II for lytic C3/C4-oxidation, C=O labeling strategy III for (oxo-)reducing ends, and CO_2_H labeling strategy IV for aldonic acid termini (see Table 3).

**Table 3:** Comparison of degradation conditions in the OBG and BBG oxidation regarding *M*_*n*_/relative theoretical reducing end content (1/*M*_*n*_) from SEC, as well as relative UPLC-MS signals of oxidized downstream and non-reducing end termini from sample preparation strategies II–IV^a^.

**BG Source**	**Reaction**	***M_*n*_* [kg/mol]**	**1/*M_*n*_* (calc.)**	**Glc red. ends (exp.)^b^**	**%REG exp./calc.^c^**	**^oxo^GlcGlc(*n*−1)^d^**	**Glc1A termini^e^**	**^5oxo^Glc reducing ends^f^**
Oat	Control	189	(0.41)	(0.52)	(100%)	(0.14)	(0.59)	(0.24)
	(a) H_2_O_2_	10.2	7.7	1.2	12%	8.3	9.2	12
	(b) AH_2_	78.1	**1**	**1**	80%	**1**	**1**	**1**
Barley	Control	243	(0.35)	(0.50)	(100%)	(0.11)	(0.50)	(0.18)
	(a) H_2_O_2_	10.0	8.5	2.0	16%	21	28	24
	(b) AH_2_	85.2	**1**	**1**	70%	**1**	**1**	**1**
Represents lytic oxidation at:			(all)	C3/C4	–	C3/C4	C1	C5

a *MS signals of lytic products only (negative mode, basic eluent). Conditions: 0.6% BG, 50 μM FeSO_4_, with (a) 100 mM H_2_O_2_ and (b) 250 μM AH_2_, 22–24°C, 24 h. All values (except M_n_ & % REG) relative to condition (b) AH_2_ (set to 1, bold). M_n_, number average molar mass; Glc, glucose; Glc1A, gluconic acid; ^5oxo^Glc, 5-ketoglucose; AH_2_, ascorbic acid; ^oxo^GlcGlc_(*n*−1)_, oxidized gluco-oligomer with new carbonyl at non-reducing end*.

b *Experimentally determined by labeling with 2-AA by reductive amination (strategy III). Total signal of reducing end species with m/z 300.11 (Glc-2AA) and m/z 462.16 (GlcGlc-2AA)*.

c *Accuracy of using 1/M_n_ for reducing end group (REG) content by dividing the relative values for Glc red. ends (exp.) by the relative values of 1/M_n_ (calc.); normalized for control (native material) = 100%*.

d *Enzyme digestion/SPE (strategy II). Total signal of major oligomeric oxo-products with C=O at the non-reducing end (n = 3, 4)*.

e *Labeling with PhNH_2_ by EDC-mediated amidation (strategy IV). Total signal of (lytic) C1 oxidation species with m/z 270.10 (Glc1A-NHPh) and m/z 432.15 (GlcGlc1A-NHPh)*.

f *Strategy III: Total signal of 5-oxo-reducing end species with m/z 281.11 and m/z 443.17 for the 2-AB tagged mono- and β-(1→3)-linked disaccharide, respectively*.

##### Comparison of reaction conditions

If oxidation under the harsh (H_2_O_2_) and mild (AH_2_) conditions only differ in the extent of oxidation, but have the same product profiles, meaning that both are based on the same mechanism with the same probabilities of initial attack regarding the different glucose carbons, then the relative amounts of detected products should be similar for each oxidation type. This was indeed the case for the termini ^oxo^GlcGlc_(*n*−1)_, Glc1A, and ^5oxo^Glc from lytic C3/C4-, C1-, and C5-oxidation, respectively (H_2_O_2_/AH_2_ ratios in the range of 8.3–12:1 for OBG; Table 3). The relative values also roughly matched the expected theoretical content of termini calculated from 1/*M*_*n*_ (7.7:1). The experimental ratio for Glc reducing ends as observed by C=O labeling, however, is with 1.2:1 much lower than expected. The same tendencies with detected Glc reducing ends < < other termini were also observed in the case of BBG oxidation (Table 3). There are (at least) two possible explanations:

- Different mechanisms: The harsher conditions with H_2_O_2_ lead to a different lytic behavior in the Fenton-induced degradation with H_2_O_2_ compared to the ascorbate-driven Fenton oxidation, for instance by forming another reactive species additionally or in changed proportions to HO^•^ (e.g., Fe^IV^ = O^2+^; Bataineh et al., 2012), which may have different reactive preferences.
- Secondary reactions: The product profiles are initially the same for the two reaction conditions (same mechanism), but the presence of large amounts of H_2_O_2_ (100 mM) leads to secondary oxidation of newly formed reducing ends, e.g., to gluconic acids, masking the originally higher reducing end values for the harsher conditions.

##### Secondary oxidation

While the first mechanistic explanation cannot be excluded, there are strong indications for secondary oxidations to be the reason for the discrepancy in H_2_O_2_/AH_2_ product amount ratios in the various product classes. Assuming ^oxo^GlcGlc_(*n*−1)_ products are mostly from lytic C3/C4 oxidation, which is (next to potential β-eliminations) arguably the main mechanism for producing new reducing ends in a 1:1 ratio under the Fenton-induced oxidative conditions with contact to O_2_ (Schuchmann and Sonntag, 1978; von Sonntag and Schuchmann, 2001), the detected relative amounts of ^oxo^GlcGlc_(*n*−1)_ under H_2_O_2_/AH_2_ of 8.3:1 for OBG should be therefore similar (if not smaller) than the observed C=O labeled reducing end content. The ^oxo^GlcGlc_(*n*−1)_ ratio is, however, 7x larger than the reducing end ratios detected as Glc-2AA/AB (1.2:1 in Table 3). Reducing ends must be therefore reacting further, e.g. in a secondary oxidation. Precedence for a preferred C1-oxidation of free reducing ends when an excess of H_2_O_2_ is present was recently reported by Mao (2016) in a glucose oxidation with only H_2_O_2_ (Glc + H_2_O_2_→Glc1A + H_2_O), where Glc1A was overwhelmingly the predominant product. Thus, determination of the new reducing end content is not suitable for the harsh conditions to deduce an estimated amount of lytic C3/C4 oxidation, while detection of ^oxo^GlcGlc_(*n*−1)_, on the other hand, is presumed to be indicative of the reducing end content prior to further oxidation to Glc1A.

The occurrence of secondary oxidations in general is also supported by the fact that roughly 10x more cross-ring cleavage products such as labeled arabinose (Ara-2AA/AB relative to Glc-2AA/AB) are found in the H_2_O_2_-compared to the AH_2_-treated samples (see Figure 7B). Arabinose is a known Ruff degradation product of Glc1A occurring when Fe^3+^/H_2_O_2_ is present (Ruff, 1898), and the proportion of this secondary product correlates well with the degree of oxidation in general, corroborating the hypothesis that an unknown portion of the Glc1A products may also originate from secondary oxidation of released reducing ends and not exclusively from direct lytic cleavage at C1, which is the primary oxidation pathway for Glc1A production as previously determined (Boulos and Nyström, 2016). It also explains why the ratio of Glc1A termini (9.2:1 for OBG; Table 3) is not roughly double the ratio of the other termini [^oxo^GlcGlc_(*n*−1)_ and ^5oxo^Glc with 8.3–12:1], which would be expected if secondary oxidation of reducing ends largely contributed to the Glc1A signal in addition to lytic C1-oxidation. The Glc1A termini, however, fall victim to secondary oxidation under the harsh conditions themselves, lowering their H_2_O_2_/AH_2_ signal ratio and producing the mentioned neutral pentose end groups detected by C=O labeling as (Glc)Ara-2AB.

Fraser-Reid et al. (1961) also noticed in the Fenton-induced degradation of methylated sugars that “high concentration of hydrogen peroxide and high temperatures favor the formation of pentoses and non-reducing acidic materials.” In irradiation experiments of Glc, Phillips et al. (1958) concluded that Ara was a degradation product of Glc1A, whereas Ery was claimed to be a primary product. Our results corroborate this assessment, as Ery exhibits a similar product profile proportion regardless of degree of oxidation, in contrast to the relative signal of Ara, which correlates with the degree of oxidation (Figure 7B). This underlines the strength of using complementary sample preparation strategies to analyze BG oxidation, as an isolated analysis of Glc1A end groups by CO_2_H labeling, for instance, might have been conducted under the assumption that they all originate directly from lytic C1-oxidation. Only comparison with the relative reaction condition values from strategies II (for lytic oxo-products) and III (for actual reducing end content) gives a complete, more refined picture including the involvement of secondary oxidation processes under the harsh conditions.

##### Implications on using 1/M_n_ for reducing end content

It is worth noting that the detected Glc-2AA/AB and GlcGlc-2AA/AB from C=O labeling strategy III represent actual carbonyls from intact reducing end groups (REG). In the literature, approximations for reducing end carbonyl amounts [C=O]_REG_ are often used based only on the determined *M*_*n*_ by means of SEC and light scattering techniques. While this is a reasonable assumption for hydrolytically degraded polysaccharides, it is not for Fenton-induced oxidations, where lytic cleavage occurs to a significant proportion also under formation of carboxylic acid end groups (e.g., Glc1A). These acidic termini are ignored in approaches where the total carbonyl content [C=O]_total_ is measured (e.g., by C=O fluorescent labeling) and the internal carbonyl content [C=O]_internal_ deduced by subtracting the calculated reducing end content [C=O]_REG_ derived from 1/*M*_*n*_ ([C=O]_internal_ = [C=O]_total_ – [C=O]_REG_) (Mäkelä et al., 2017). Also completely ignored in such calculations are the non-reducing end carbonyl groups from lytic C3/C4 oxidation, which are lumped together with the “internal C=Os.”

To determine how substantial the error is when using 1/*M*_*n*_ for reducing end group content in oxidized materials, we calculated the percentage of experimentally determined actual Glc reducing ends to the relative theoretical downstream ends 1/*M*_*n*_. For this comparison, the results were normalized by setting the native materials = 100%, assuming all downstream ends in the native material being Glc reducing ends, which is a reasonable approximation. While the percentages for the mild conditions were with 70–80% relatively close to the theoretical 100%, the harsh oxidation exhibited a large difference (Table 3; column %REG). Only 12–16% of the calculated downstream ends were actually Glc reducing ends—the rest were oxidized termini such as Glc1A. The results did not significantly change when labeled Ara, Ery, and ^5oxo^Glc reducing ends were also included in the calculation (not shown). Hence, 1/*M*_*n*_ is in no way proportional to the actual reducing end carbonyl amount for the Fenton-induced oxidation, and gives faulty, grossly underestimated results when used to infer the internal carbonyl content. Our presented C=O labeling strategy III with enzymatic digestion/SPE and UPLC-MS detection, on the other hand, allows for the determination of actual reducing end contents and hence a more accurate picture of the oxidation profile from the Fenton-induced degradation of β-glucan.

## Conclusions

The presented four complementary sample preparation strategies for analysis of cereal BG oxidation by UPLC-MS/MS were demonstrated to allow access to information about different oxidation product classes. Together, they provide a complete picture of the Fenton-induced degradation, including the identification of some secondary oxidation processes. The use of hydrophilic interaction chromatography and eluent systems specific for retention of neutral or acidic products facilitated product identification in direct-SPE strategy I and enzyme digestion/SPE strategy II, with MS/MS of deprotonated species allowing for the localization of the oxidation site and the β-(1→3)-bond in the oligo-product. Observed DP3/DP4 product ratios as well as labeled mono- to disaccharide ratios are in accordance with theoretical considerations of oxidation pathways and enzyme digestion, corroborating the random nature of Fenton-induced oxidations for C1, C3&4, and C6-oxidation.

Relative amounts of labeled Glc reducing ends revealed that the common practice of using 1/*M*_*n*_ to calculate the reducing end content is a poor choice for polysaccharides oxidized under Fenton-conditions, with harsher oxidations leading to an overestimation of reducing ends by nearly one order of magnitude when using 1/*M*_*n*_ due to secondary oxidations. The presented C=O labeling strategy III with enzymatic digestion/SPE and UPLC-MS, on the other hand, allows for the accurate determination of actual reducing ends, and reveals in what form they occur (Glc, Ara, Ery).

The use of enzyme digestions specific to cereal BG simplifies the product profiles and allows for the distinction of mixed-linkage BG oxidation from other polysaccharides and even other glucans (e.g., starch, cellulose) as showcased in the co-oxidation of BG and corn starch. To the best of our knowledge, this is the first reported method where specificity for BG oxidation was demonstrated in a mixture without the need for prior extensive extraction to obtain the BG polymer free of other polysaccharide in order to detect its oxidation products. In addition to their specificity for BG oxidation, the enzyme digestion/SPE, carbonyl- and carboxylate-labeling methods were demonstrated to be sensitive enough to detect mild oxidation induced by the ascorbate-driven Fenton reaction relevant to food processing or storage (strategies II–IV).

This exploratory study serves as a proof of concept for the various possibilities to detect BG oxidation products by UPLC-MS/MS in order to gain detailed and complete structural information on oxidation sites and insights to the mechanistic aspects of the degradation through comparison of signal ratios. Future studies based on the presented analytical methods, for instance with isotopic tagging techniques of standards in the C=O and CO_2_H labeling strategies III & IV, will correct for varying sample preparation efficiencies and for potential matrix effects regarding ionization. This will allow absolute quantification by MS of BG oxidation products in food processing on a detailed level that goes beyond simple functional group quantification, opening doors to new realms of understanding regarding BG's structure-function relationships.

## Author contributions

LN and SB conceived the idea and designed the work. SB performed all experiments, data analysis, and interpretation. Both authors contributed to the manuscript and approved the final version.

### Conflict of interest statement

The authors declare that the research was conducted in the absence of any commercial or financial relationships that could be construed as a potential conflict of interest.

## References

[B1] Airianah O. B. Vreeburg R. A. M. Fry S. C. (2016). Pectic polysaccharides are attacked by hydroxyl radicals in ripening fruit: evidence from a fluorescent fingerprinting method. Ann. Bot. 117, 441–455. 10.1093/aob/mcv192 26865506 PMC4765547

[B2] Anumula K. R. Dhume S. T. (1998). High resolution and high sensitivity methods for oligosaccharide mapping and characterization by normal phase high performance liquid chromatography following derivatization with highly fluorescent anthranilic acid. Glycobiology 8, 685–694. 10.1093/glycob/8.7.685 9621109

[B3] Barras D. R. Moore A. E. Stone B. A. (1969). Enzyme-substrate relationships among β-glucan hydrolases, in Cellulases and Their Applications, ed Gould R. F. (Washington, DC: American Chemical Society), 105–138.

[B4] Bataineh H. Pestovsky O. Bakac A. (2012). pH-induced mechanistic changeover from hydroxyl radicals to iron(iv) in the Fenton reaction. Chem. Sci. 3, 1594–1599. 10.1039/c2sc20099f

[B5] Baxter E. W. Reitz A. B. (1994). Expeditious synthesis of Aza sugars by the double reductive amination of dicarbonyl sugars. J. Org. Chem. 59, 3175–3185. 10.1021/jo00090a040

[B6] Bigge J. C. Patel T. P. Bruce J. A. Goulding P. N. Charles S. M. Parekh R. B. (1995). Nonselective and efficient fluorescent labeling of glycans using 2-amino benzamide and anthranilic acid. Analyt. Biochem. 230, 229–238. 10.1006/abio.1995.1468 7503412

[B7] Boulos S. Nyström L. (2016). UPLC-MS/MS investigation of β-glucan oligosaccharide oxidation. Analyst 141, 6533–6548. 10.1039/C6AN01125J 27868112

[B8] Cumpstey I. (2013). Chemical modification of polysaccharides. ISRN Org. Chem. 2013:417672. 10.1155/2013/417672 24151557 PMC3787328

[B9] Domon B. Costello C. (1988). A systematic nomenclature for carbohydrate fragmentations in FAB-MS/MS spectra of glycoconjugates. Glycoconj. J. 5, 397–409. 10.1007/BF01049915

[B10] EFSA (2011). EFSA Panel on dietetic products, nutrition and allergies (NDA). Scientific opinion on the substantiation of health claims related to beta-glucans from oats and barley and maintenance of normal blood LDL-cholesterol concentrations (ID 1236, 1299), increase in satiety leading to a reduction in energy intake (ID 851, 852), reduction of post-prandial glycaemic responses (ID 821, 824), and “digestive function” (ID 850) pursuant to Article 13(1) of Regulation (EC) No 1924/2006. EFSA J. 9:2207 10.2903/j.efsa.2011.2207

[B11] Faure A. M. Knüsel R. Nyström L. (2013a). Effect of the temperature on the degradation of β-glucan promoted by iron(II). Bioact. Carbohyd. Diet. Fibre 2, 99–107. 10.1016/j.bcdf.2013.09.003

[B12] Faure A. M. Münger L. H. Nyström L. (2012). Potential inhibitors of the ascorbate-induced beta-glucan degradation. Food Chem. 134, 55–63. 10.1016/j.foodchem.2012.02.055

[B13] Faure A. M. Sànchez-Ferrer A. Zabara A. Andersen M. L. Nyström L. (2014). Modulating the structural properties of beta-D-glucan degradation products by alternative reaction pathways. Carbohyd. Polym. 99, 679–686. 10.1016/j.carbpol.2013.08.022 24274558

[B14] Faure A. M. Werder J. Nyström L. (2013b). Reactive oxygen species responsible for beta-glucan degradation. Food Chem. 141, 589–596. 10.1016/j.foodchem.2013.02.096 23768398

[B15] FDA (2009). 21 CFR 101.81 - Health claims: Soluble fiber from certain foods and risk of coronary heart disease (CHD), in Regulatory Information Title 21 (Chapter I, Subchapter B, Part 101, Subpart E, Section 101.81), 143–148.

[B16] Fenton H. J. H. (1894). LXXIII.-Oxidation of tartaric acid in presence of iron. J. Chem. Soc. Trans. 65, 899–910.

[B17] Fraser-Reid B. Perry M. B. Jones J. K. N. (1961). Demethylation of sugars with hydrogen peroxide. Can. J. Chem. 39, 555 10.1139/v61-067

[B18] Frommhagen M. van Erven G. Sanders M. van Berkel W. J. H. Kabel M. A. Gruppen H. (2017). RP-UHPLC-UV-ESI-MS/MS analysis of LPMO generated C4-oxidized gluco-oligosaccharides after non-reductive labeling with 2-aminobenzamide. Carbohydr. Res. 448, 191–199. 10.1016/j.carres.2017.03.006 28302276

[B19] Fry S. C. Nesselrode B. H. Miller J. G. Mewburn B. R. (2008). Mixed-linkage (1→3,1→4)-β-d-glucan is a major hemicellulose of Equisetum (horsetail) cell walls. New Phytol. 179, 104–115. 10.1111/j.1469-8137.2008.02435.x 18393951

[B20] Huang Z. B. Prickett T. Potts M. Helm R. F. (2000). The use of the 2-aminobenzoic acid tag for oligosaccharide gel electrophoresis. Carbohydr. Res. 328, 77–83. 10.1016/S0008-6215(00)00045-8 11005578

[B21] Iurlaro A. Dalessandro G. Piro G. Miller J. G. Fry S. C. Lenucci M. S. (2014). Evaluation of glycosidic bond cleavage and formation of oxo groups in oxidized barley mixed-linkage beta-glucans using tritium labelling. Food Res. Int. 66, 115–122. 10.1016/j.foodres.2014.09.008

[B22] Kivelä R. Gates F. Sontag-Strohm T. (2009a). Degradation of cereal beta-glucan by ascorbic acid induced oxygen radicals. J. Cereal Sci. 49, 1–3. 10.1016/j.jcs.2008.09.003

[B23] Kivelä R. Henniges U. Sontag-Strohm T. Potthast A. (2012). Oxidation of oat beta-glucan in aqueous solutions during processing. Carbohydr. Polym. 87, 589–597. 10.1016/j.carbpol.2011.08.028 34663008

[B24] Kivelä R. Nyström L. Salovaara H. Sontag-Strohm T. (2009b). Role of oxidative cleavage and acid hydrolysis of oat beta-glucan in modelled beverage conditions. J. Cereal Sci. 50, 190–197. 10.1016/j.jcs.2009.04.012

[B25] Kivelä R. Pitkanen L. Laine P. Aseyev V. Sontag-Strohm T. (2010). Influence of homogenisation on the solution properties of oat beta-glucan. Food Hydrocoll. 24, 611–618. 10.1016/j.foodhyd.2010.02.008

[B26] Kivelä R. Sontag-Strohm T. Loponen J. Tuomainen P. Nyström L. (2011). Oxidative and radical mediated cleavage of beta-glucan in thermal treatments. Carbohydr. Polym. 85, 645–652. 10.1016/j.carbpol.2011.03.026

[B27] Lane C. F. (1975). Sodium cyanoborohydride - a highly selective reducing agent for organic functional groups. Synthesis 1975, 135–146. 10.1055/s-1975-23685

[B28] Lazaridou A. Biliaderis C. G. (2007). Molecular aspects of cereal beta-glucan functionality: physical properties, technological applications and physiological effects. J. Cereal Sci. 46, 101–118. 10.1016/j.jcs.2007.05.003

[B29] Lee K. Y. Park S. Y. Lee H. G. (2011). Effect of oat beta-glucan and its oxidised derivative on the quality characteristics of sponge cake. Int. J. Food Sci. Tech. 46, 2663–2668. 10.1111/j.1365-2621.2011.02798.x

[B30] Leijdekkers A. G. M. Sanders M. G. Schols H. A. Gruppen H. (2011). Characterizing plant cell wall derived oligosaccharides using hydrophilic interaction chromatography with mass spectrometry detection. J. Chromatogr. A 1218, 9227–9235. 10.1016/j.chroma.2011.10.068 22099219

[B31] Li G. Li L. Xue C. Middleton D. Linhardt R. J. Avci F. Y. (2015). Profiling pneumococcal type 3-derived oligosaccharides by high resolution liquid chromatography-tandem mass spectrometry. J. Chromatogr. A 1397, 43–51. 10.1016/j.chroma.2015.04.009 25913329 PMC4426055

[B32] Lindsay S. E. Fry S. C. (2007). Redox and wall-restructuring, in The Expanding Cell, eds Verbelen J. -P. Vissenberg K. (Berlin; Heidelberg, New York, NY: Springer), 159–190.

[B33] Maina N. H. Juvonen M. Domingues R. M. Virkki L. Jokela J. Tenkanen M. (2013). Structural analysis of linear mixed-linkage glucooligosaccharides by tandem mass spectrometry. Food Chem. 136, 1496–1507. 10.1016/j.foodchem.2012.09.075 23194554

[B34] Mäkelä N. Sontag-Strohm T. Maina N. H. (2015). The oxidative degradation of barley β-glucan in the presence of ascorbic acid or hydrogen peroxide. Carbohydr. Polym. 123, 390–395. 10.1016/j.carbpol.2015.01.037 25843872

[B35] Mäkelä N. Sontag-Strohm T. Schiehser S. Potthast A. Maaheimo H. Maina N. H. (2017). Reaction pathways during oxidation of cereal β-glucans. Carbohydr. Polym. 157, 1769–1776. 10.1016/j.carbpol.2016.11.060 27987894

[B36] Malawer E. G. Senak L. (2003). Introduction to size exclusion chromatography, in Handbook of Size Exclusion Chromatography and Related Techniques, ed Wu C.-S. (New York, NY; Basel: CRC Press).

[B37] Mao Y.-M. (2016). Preparation of gluconic acid by oxidation of glucose with hydrogen peroxide. J. Food Process. Preserv. 41:e12742 10.1111/jfpp.12742

[B38] McCleary B. V. Harrington J. (1988). Purification of β-d-glucosidase from *Aspergillus niger*, in Methods in Enzymology, eds Wood W. A. Kellogg S. T. (Cambridge, MA: Academic Press), 575–583.

[B39] Michels A. J. Frei B. (2013). Myths, artifacts, and fatal flaws: identifying limitations and opportunities in vitamin C research. Nutrients 5, 5161–5192. 10.3390/nu5125161 24352093 PMC3875932

[B40] Ovalle R. Soll C. E. Lim F. Flanagan C. Rotunda T. Lipke P. N. (2001). Systematic analysis of oxidative degradation of polysaccharides using PAGE and HPLC–MS. Carbohydr. Res. 330, 131–139. 10.1016/S0008-6215(00)00262-7 11217956

[B41] Packer N. H. Lawson M. A. Jardine D. R. Redmond J. W. (1998). A general approach to desalting oligosaccharides released from glycoproteins. Glycoconj. J. 15, 737–747. 10.1023/A:1006983125913 9870349

[B42] Park J. S. B. Wood P. M. Gilbert B. C. Whitwood A. C. (1999). EPR Evidence for hydroxyl- and substrate-derived radicals in Fe(II)-oxalate/hydrogen peroxide reactions. The importance of the reduction of Fe(III)-oxalate by oxygen-conjugated radicals to regenerate Fe(II) in reactions of carbohydrates and model compounds. J. Chem. Soc. Perk. T 2, 923–931.

[B43] Park S. Y. Bae I. Y. Lee S. Lee H. G. (2009). Physicochemical and hypocholesterolemic characterization of oxidized oat beta-glucan. J. Agric. Food Chem. 57, 439–443. 10.1021/jf802811b 19119839

[B44] Phillips G. O. Moody G. J. Mattok G. L. (1958). 710. Radiation chemistry of carbohydrates. Part I. Action of ionising radiation on aqueous solutions of D-glucose. J. Chem. Soc. 3522–3534.

[B45] Redmond J. W. Packer N. H. (1999). The use of solid-phase extraction with graphitised carbon for the fractionation and purification of sugars. Carbohydr. Res. 319, 74–79. 10.1016/S0008-6215(99)00130-5

[B46] Regand A. Chowdhury Z. Tosh S. M. Wolever T. M. S. Wood P. (2011). The molecular weight, solubility and viscosity of oat beta-glucan affect human glycemic response by modifying starch digestibility. Food Chem. 129, 297–304. 10.1016/j.foodchem.2011.04.053 30634230

[B47] Regand A. Tosh S. M. Wolever T. M. S. Wood P. J. (2009). Physicochemical properties of beta-glucan in differently processed oat foods influence glycemic response. J. Agric. Food Chem. 57, 8831–8838. 10.1021/jf901271v 19728711

[B48] Ruff O. (1898). Ueber die verwandlung der d-gluconsäure in d-arabinose. Ber. Dtsch. Chem. Ges. 31, 1573–1577. 10.1002/cber.18980310250

[B49] Saha A. K. Brewer C. F. (1994). Determination of the concentrations of oligosaccharides, complex type carbohydrates, and glycoproteins using the phenol-sulfuric acid method. Carbohydr. Res. 254, 157–167. 10.1016/0008-6215(94)84249-3 8180982

[B50] Schuchmann M. N. Sonntag C. V. (1977). Radiation-chemistry of carbohydrates.14. Hydroxyl radical induced oxidation of D-glucose in oxygenated aqueous-solution. J. Chem. Soc. Perk. T 2, 1958–1963.

[B51] Schuchmann M. N. Sonntag C. V. (1978). Effect of oxygen on OH-radical-induced scission of glycosidic linkage of cellobiose. Int. J. Radiat. Biol. 34, 397–400. 10.1080/09553007814551051 309876

[B52] Simmons T. J. Uhrín D. Gregson T. Murray L. Sadler I. H. Fry S. C. (2013). An unexpectedly lichenase-stable hexasaccharide from cereal, horsetail and lichen mixed-linkage β-glucans (MLGs): implications for MLG subunit distribution. Phytochemistry 95, 322–332. 10.1016/j.phytochem.2013.08.003 24025426

[B53] Simões J. Moreira A. S. P. da Costa E. Evtyugin D. Domingues P. Nunes F. M.. (2016). Oxidation of amylose and amylopectin by hydroxyl radicals assessed by electrospray ionisation mass spectrometry. Carbohydr. Polym. 148, 290–299. 10.1016/j.carbpol.2016.03.034 27185142

[B54] Sonntag C. V. Dizdaroglu M. Schultefrohlinde D. (1976). Radiation-chemistry of carbohydrates.8. gamma-radiolysis of cellobiose in N2O-saturated aqueous-solution. 2. quantitative measurements - mechanisms of radical-induced scission of glycosidic linkage. J. Chem. Sci. 31, 857–864.

[B55] Tosh S. M. Brummer Y. Miller S. S. Regand A. Defelice C. Duss R.. (2010). Processing affects the physicochemical properties of beta-glucan in oat bran cereal. J. Agric. Food Chem. 58, 7723–7730. 10.1021/jf904553u 20527967

[B56] Tosh S. M. Brummer Y. Wood P. J. Wang Q. Weisz J. (2004). Evaluation of structure in the formation of gels by structurally diverse (1→3)(1→4)-beta-D-glucans from four cereal and one lichen species. Carbohydr. Polym. 57, 249–259. 10.1016/j.carbpol.2004.05.009

[B57] Valeur E. Bradley M. (2009). Amide bond formation: beyond the myth of coupling reagents. Chem. Soc. Rev. 38, 606–631. 10.1039/B701677H 19169468

[B58] von Sonntag C. Schuchmann H. P. (2001). Carbohydrates, in Studies in Physical and Theoretical Chemistry, eds Charles D. J. Rao B. S. M. (Amsterdam: Elsevier), 481–511.

[B59] Vreeburg R. A. M. Airianah O. B. Fry S. C. (2014). Fingerprinting of hydroxyl radical-attacked polysaccharides by N-isopropyl-2-aminoacridone labelling. Biochem. J. 463, 225–237. 10.1042/BJ20140678 25072268 PMC4170706

[B60] Wang Y. J. Zhan R. Sontag-Strohm T. Maina N. H. (2017). The protective role of phytate in the oxidative degradation of cereal beta-glucans. Carbohydr. Polym. 169, 220–226. 10.1016/j.carbpol.2017.04.016 28504139

[B61] Westereng B. Agger J. W. Horn S. J. Vaaje-Kolstad G. Aachmann F. L. Stenstrom Y. H.. (2013). Efficient separation of oxidized cello-oligosaccharides generated by cellulose degrading lytic polysaccharide monooxygenases. J. Chromatogr. A 1271, 144–152. 10.1016/j.chroma.2012.11.048 23246088

[B62] Westereng B. Arntzen M. O. Aachmann F. L. Varnai A. Eijsink V. G. Agger J. W. (2016). Simultaneous analysis of C1 and C4 oxidized oligosaccharides, the products of lytic polysaccharide monooxygenases acting on cellulose. J. Chromatogr. A 1445, 46–54. 10.1016/j.chroma.2016.03.064 27059395

[B63] Wolever T. M. S. Tosh S. M. Gibbs A. L. Brand-Miller J. Duncan A. M. Hart V.. (2010). Physicochemical properties of oat beta-glucan influence its ability to reduce serum LDL cholesterol in humans: a randomized clinical trial. Am. J. Clin. Nutr. 92, 723–732. 10.3945/ajcn.2010.29174 20660224

[B64] Wood P. J. (2010). Oat and rye beta-glucan: properties and function. Cereal Chem. 87, 315–330. 10.1094/CCHEM-87-4-0315

[B65] Wood P. J. Beer M. U. Butler G. (2000). Evaluation of role of concentration and molecular weight of oat beta-glucan in determining effect of viscosity on plasma glucose and insulin following an oral glucose load. Br. J. Nutr. 84, 19–23. 10.1017/S0007114500001185 10961156

[B66] Woodward R. B. Hoffmann R. (1969). The conservation of orbital symmetry. Angew. Chem. Int. Ed. Engl. 8, 781–853. 10.1002/anie.196907811

[B67] Yang W. C. Sedlak M. Regnier F. E. Mosier N. Ho N. Adamec J. (2008). Simultaneous quantification of metabolites involved in central carbon and energy metabolism using reversed-phase liquid chromatography-mass spectrometry and *in vitro* C-13 labeling. Analyt. Chem. 80, 9508–9516. 10.1021/ac801693c 19007244

[B68] Zhao X. Yang B. Li L. Zhang F. Linhardt R. J. (2013). On-line separation and characterization of hyaluronan oligosaccharides derived from radical depolymerization. Carbohydr. Polym. 96, 503–509. 10.1016/j.carbpol.2013.04.009 23768593 PMC3711257

