# Supplementary material for: Complementary Sample Preparation Strategies for Analysis of Cereal β-Glucan Oxidation Products by UPLC-MS/MS
Source: Front Chem. 2017 Nov 2;5:90. doi: 10.3389/fchem.2017.00090 (PMC5673685; doi:10.3389/fchem.2017.00090)
Supplement: Supplementary file 1 [file DataSheet1.pdf]

Supplementary Material

Complementary sample preparation strategies for analysis of cereal β-glucan oxidation products by UPLC-MS/MS

Samy Boulos, Laura Nyström*

Laboratory of Food Biochemistry, Institute of Food, Nutrition and Health, Department of Health Sciences and Technology, ETH Zurich, 8092 Zurich, Switzerland.

*** Correspondence:** Laura Nyström: laura.nystroem@hest.ethz.ch

Supplementary Tables and Figures

Figure S1	Page	2
Figure S2	Page	3
Figure S3	Page	4
Figure S4	Page	5
Figure S5	Page	6
Figure S6	Page	7
Figure S7	Page	8
Figure S8	Page	9
Figure S9	Page	10
Figure S10	Page	11
Figure S11	Page	12
Table S1	Page	13
References	Page	14

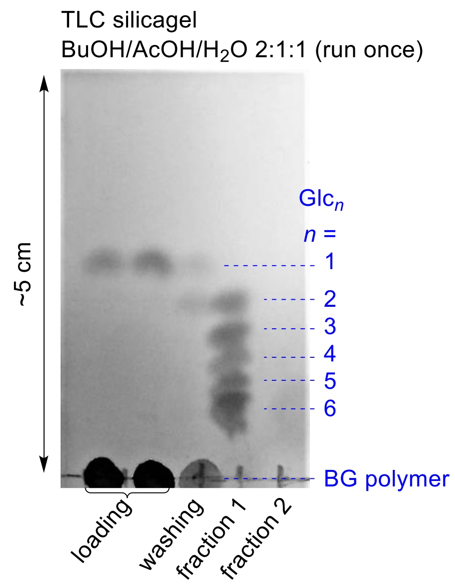

Figure S1 Thin layer chromatography (TLC) of a control experiment to establish behavior of polymeric β-glucan (BG) and oligosaccharides on graphitized carbon solid phase extraction (SPE). Preconditioned SPE cartridge (see experimental section for details) was loaded with two portions (each 2.5 mL) of 0.6% barley BG spiked with maltooligosaccharides Glc_n ($n = 1$ (1 mM); $n = 2$, 3 (each 0.4 mM); $n = 4–6$ (each 0.2 mM)), the SPE then washed with H_2O (3 mL), and eluted with 1:3 ACN/H_2O (2 × 2.5 mL; fraction 1 & 2). Solutions collected during loading (two spots), washing (1 spot), and elution (2 spots) were applied on the TLC plate (~3 µL each), and the spots made visible after development (BuOH/AcOH/H_2O 2:1:1) by dipping in an ethanolic solution of 5% (v/v) H_2SO_4 + 0.7% (w/v) 4-cumylphenol and heating for 15 min at 120°C. The TLC plate clearly shows how polymeric BG is not retained and breaks through during loading and washing (intense spot at baseline). The same is the case for glucose ($n = 1$). The rest of the spiked oligosaccharides with $n = 2–6$ are retained (small losses during washing for $n = 2$) and need aqueous ACN to fully elute in fraction 1 (no spots in fraction 2). BuOH, *n*-butanol; AcOH, acetic acid.

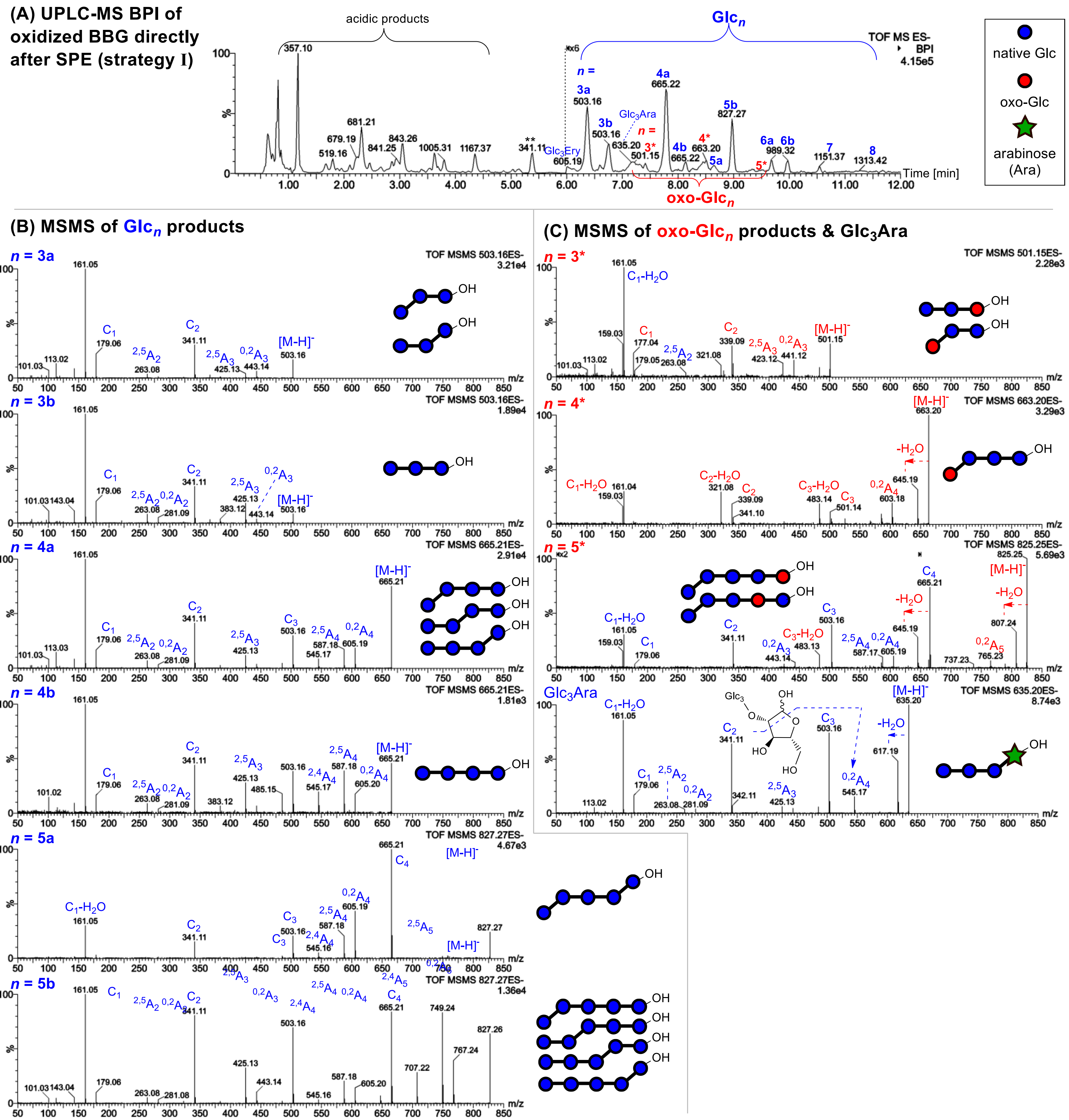

Figure S2 UPLC-MS/MS of released oligomers observed from oxidized BBG (100 mM H_2O_2, 50 µM $FeSO_4$) after direct SPE (strategy I). **(A)** Base peak ion chromatogram (BPI) using negative mode and an ACN/H_2O gradient up to 50% H_2O (0.1% NH_3 additive). **(B)** MS/MS of released Glc$_n$ and **(C)** MS/MS of oxo-Glc$_n$ species (plus the cross-ring cleavage product Glc$_3$Ara), with their proposed main oligosaccharide structures in symbolic representation, based on retention time behavior and MS/MS patterns. **, disaccharide signal from catalase material.

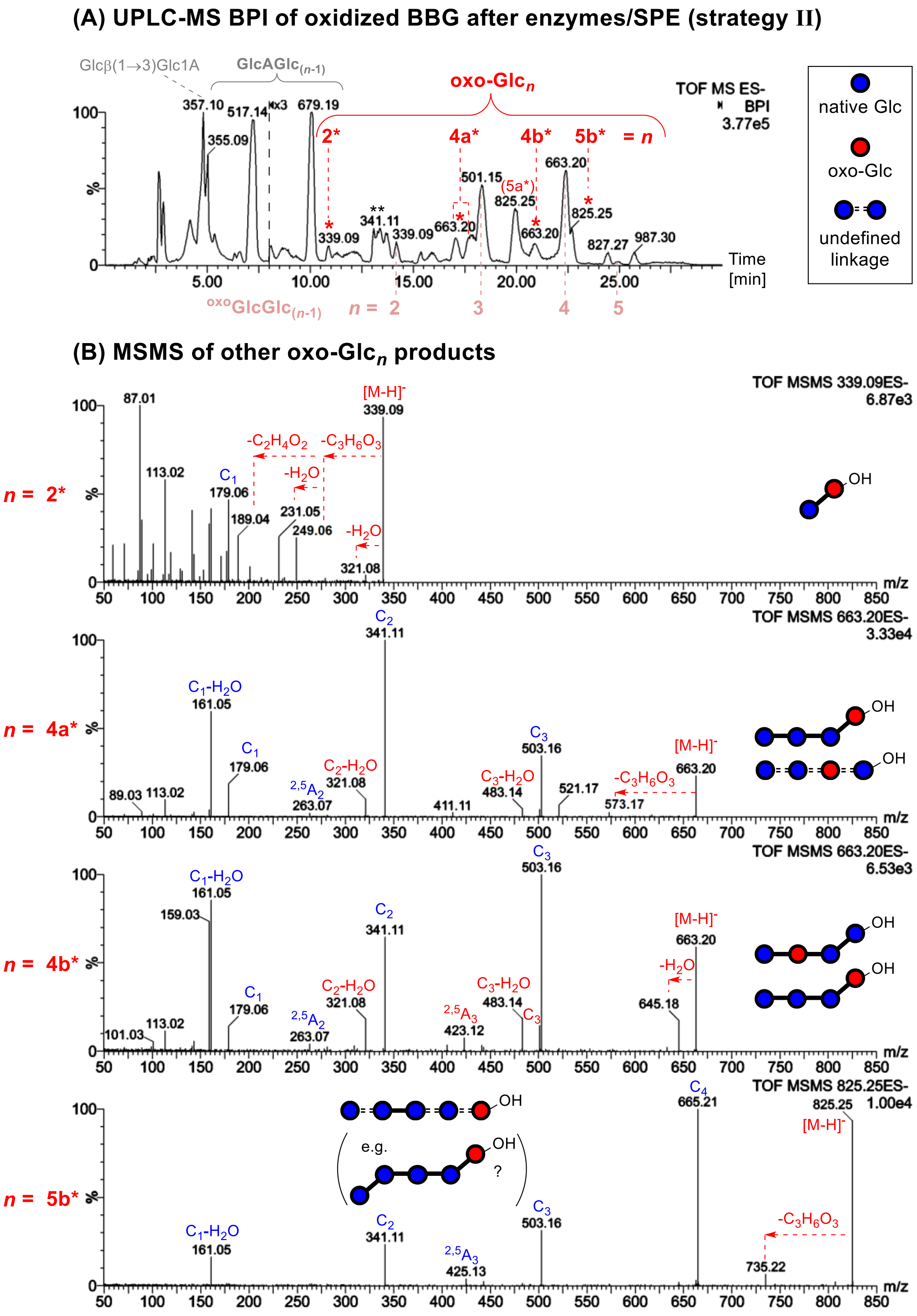

Figure S3 UPLC-MS/MS of released oxo-Glc$_n$ species with C=O located somewhere else than the non-reducing end as observed from oxidized BBG (100 mM H_2O_2, 50 µM $FeSO_4$) after enzymatic treatment/SPE (strategy II; 10x concentrated by evaporation under a stream of N_2). **(A)** Base peak ion chromatogram (BPI) using negative ion mode, a slower aqueous ACN gradient (0.17 mL/min) with 15 cm BEH amide column (doubling the retention time compared to **Figure 5a** in the manuscript), and basic eluent (0.1% NH_3 additive). **(B)** MS/MS of oxo-Glc$_n$ species. (For MS/MS of ^{oxo}GlcGlc$_{(n\text{-}1)}$ (C=O at non-reducing end) and of the (5a*) species (= oxo-Glc$_5$), see **Figure 5a/b** in the manuscript; for XICs of each oxo-Glc$_n$, see **Figure S4**).

Comparison: Harsh vs. mild oxidation of BBG after enzyme digestion/SPE (strategy II)

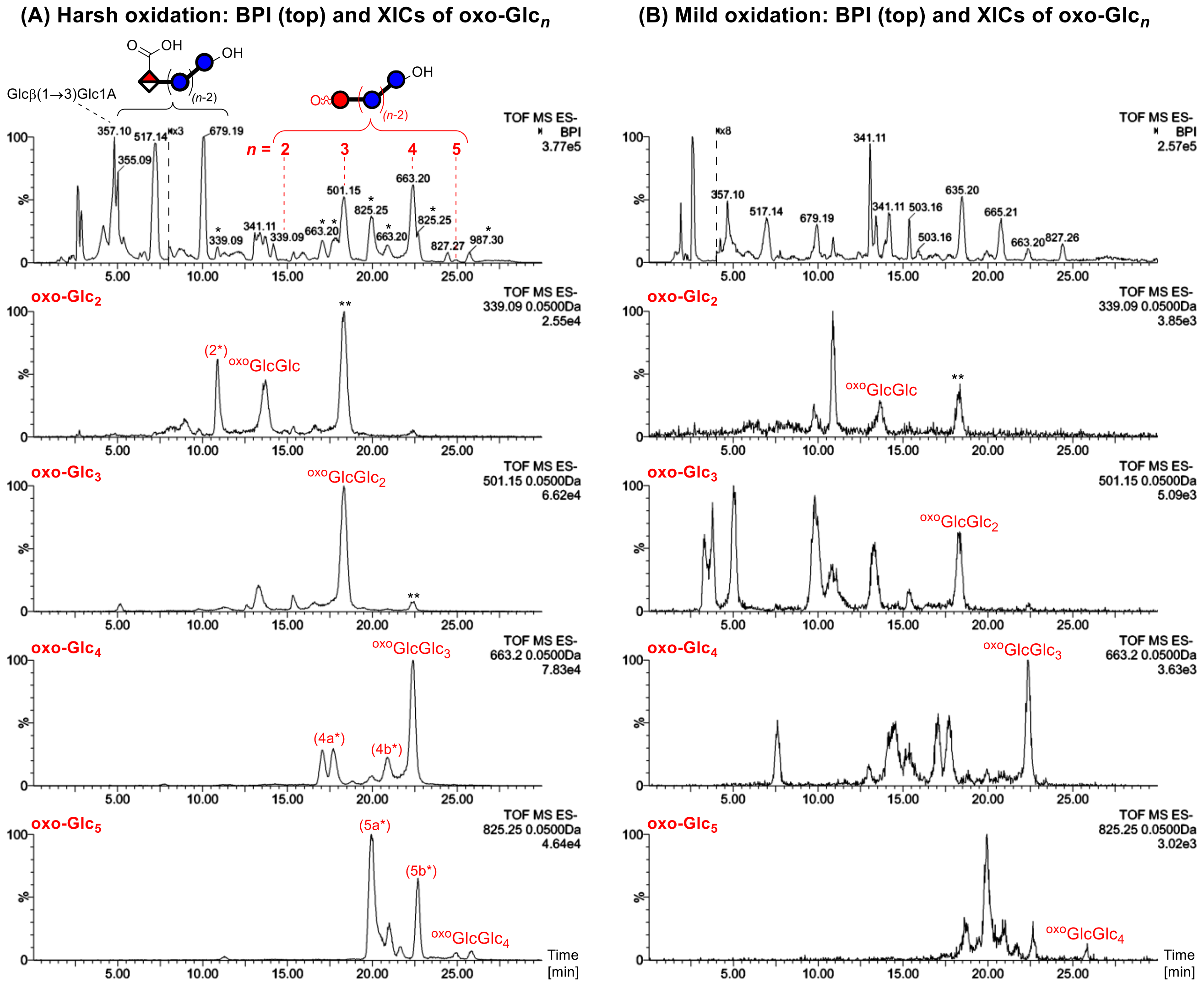

Figure S4 Comparison of UPLC-MS base peak ion (BPI) and extracted ion chromatograms (XIC) from **(A)** harsh (100 mM H_2O_2) and **(B)** mild (250 µM AH_2) oxidation of BBG after lichenase+β-glucosidase treatment/SPE (strategy II; 10x concentrated by evaporation under a stream of N_2). Negative ion mode, a slower aqueous ACN gradient (0.17 mL/min) with 15 cm BEH amide column, and basic eluent (0.1% NH_3 additive) were used. The peaks are labeled with their base peak *m*/*z*. Note that the mild oxidation conditions lead to a different product profile of oxo-Glc$_n$ species with ^{oxo}GlcGlc$_{(n\text{-}1)}$ not being the predominant product as is the case for n = 3,4 under the harsh conditions. This phenomenon is subject to further investigation. The BPI of the mild oxidation (B) shows native Glc$_n$ peaks (*m*/*z* 341, 503, 665, 827), all of which also occur in the non-oxidized control in the same proportions (whereas no oxo-Glc$_n$ signals in control), and might be residues of DP3–5 (from lichenase treatment) that were not fully hydrolyzed by β-glucosidase. The sharp *m*/*z* 341 peak (13 min) originates from the catalase material (disaccharide). *, oxo-Glc$_n$ isomers with the carbonyl not at the non-reducing end. **, in-source fragmentation peak of the respective n+1 species.

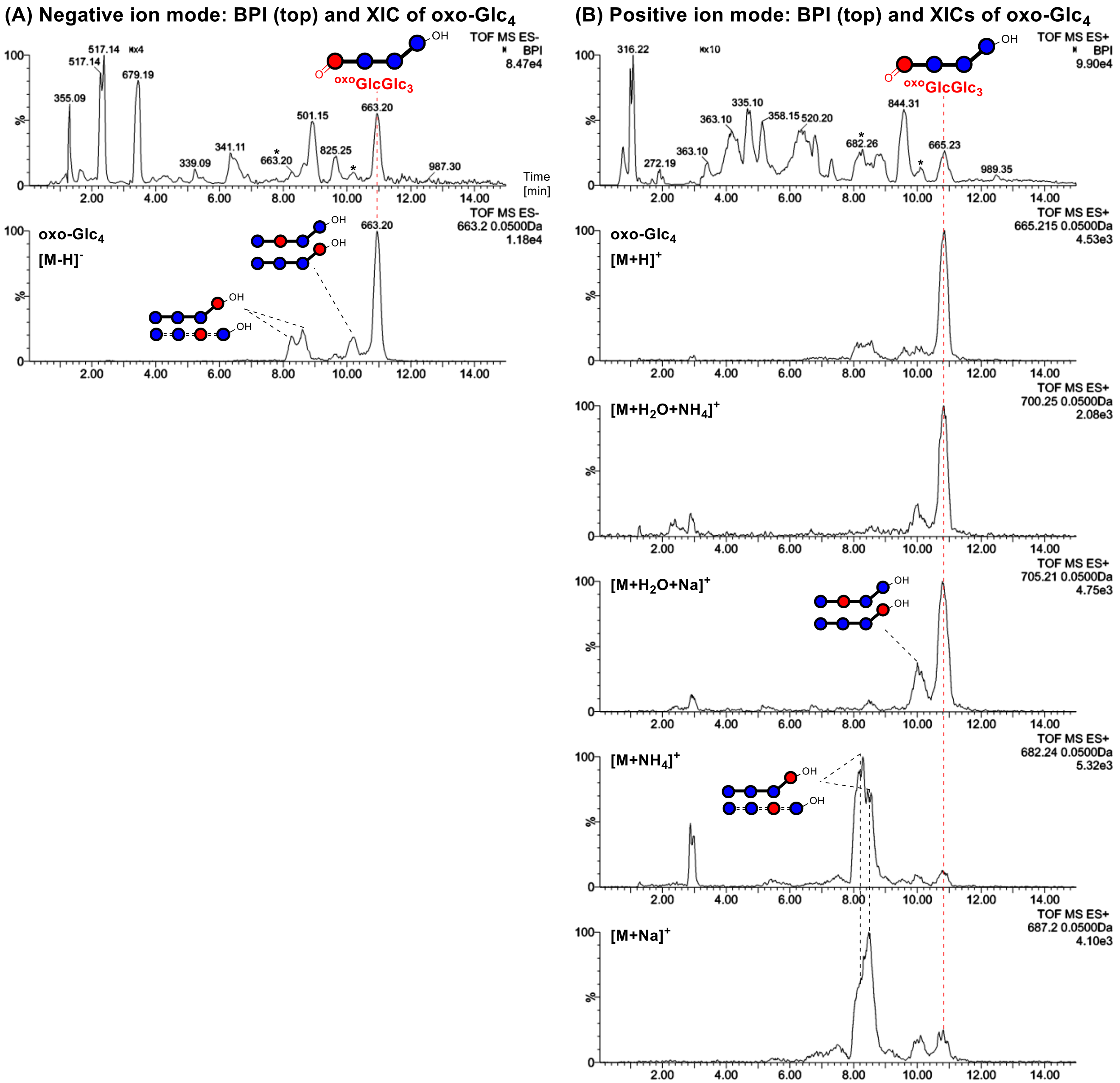

Figure S5 Comparison of (A) negative and (B) positive ion mode in the UPLC-MS analysis of BBG oxidation products after lichenase & β-glucosidase/SPE treatment (strategy II) with the respective base peak ion chromatograms (BPI) and extracted ion chromatograms (XIC) of oxo-Glc_4 as example. Note that in the positive mode, isomeric oxo-Glc_4 products have different preferences regarding ionization, with the main $^{oxo}GlcGlc_3$ product ionizing preferably as $[M+H]^-$ or $[M+H_2O+NH_4/Na]^+$, in agreement with the carbonyl-hydrate (geminal diol) equilibrium of the oxo-group ($R_2C{=}O + H_2O = R_2C(OH)_2$). Other oxo-$Glc_4$ isomers with mid-chain oxo-groups (labeled with * in the BPIs) preferably ionize as ammonium or sodium adducts in the positive ion mode.

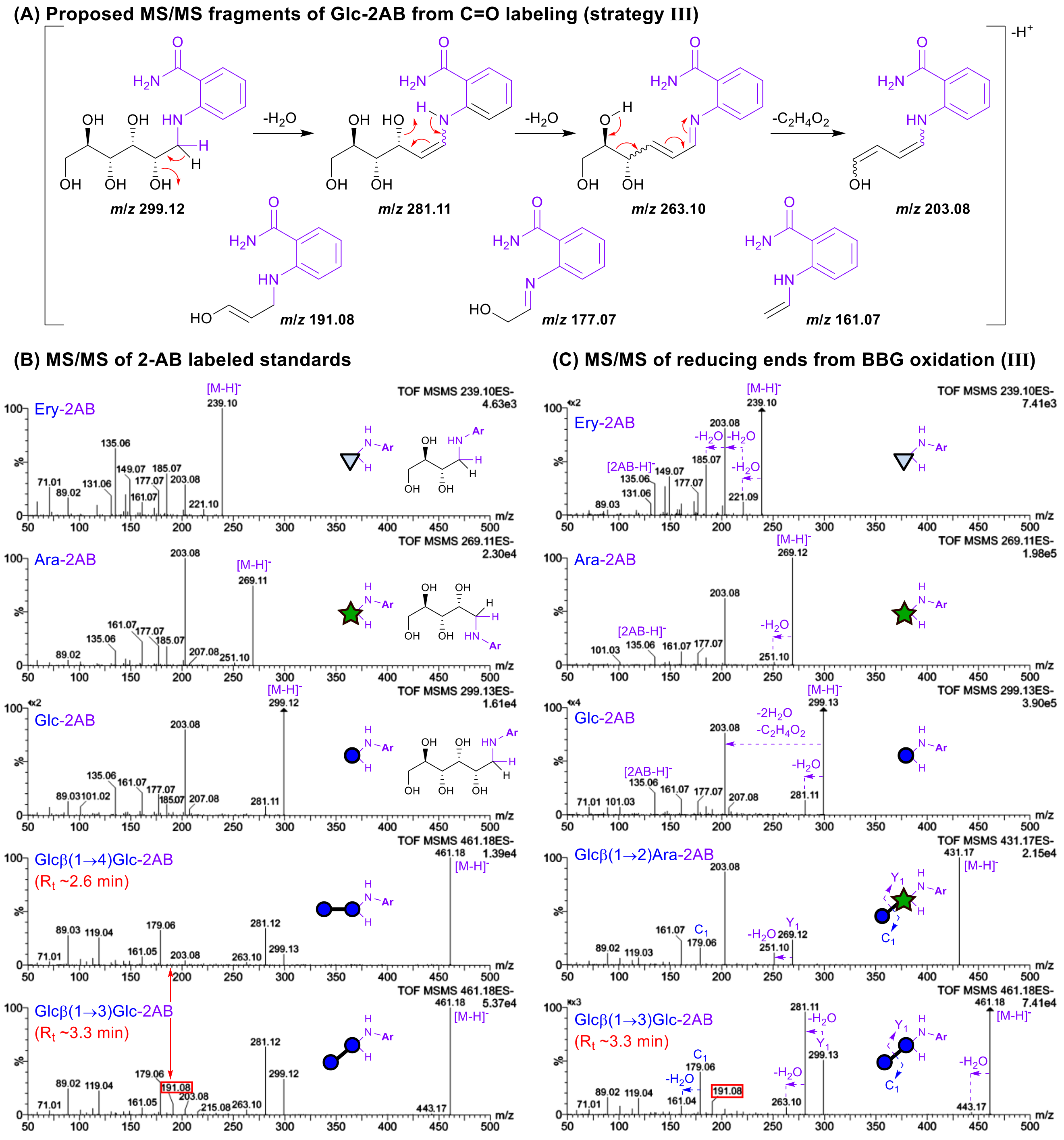

Figure S6 (A) Collision induced dissociation (CID) of Glc-2AB from reductive amination strategy III: Proposed structures for the observed most prominent MS/MS fragments. MS/MS spectra of **(B)** 2-AB labeled standards, and **(C)** of 2-AB labeled reducing termini from harsh BBG oxidation after enzyme treatment/SPE (fraction 2; see **Figure 7b** for BPI). Fragments are labeled for (C), with labels in purple containing the 2-AB moiety, while labels in blue do not. Differences in relative fragment intensities of Ery- and Ara-2AB between standards and BBG oxidation products might originate from Ara and Ery being different isobaric pentoses or tetroses, respectively, e.g. formed through epimerization. Oxidation product GlcGlc-2AB could be identified to be the β-(1→3)-linked isomer due to the observed MS/MS fragment *m/z* 191 and matching retention time (R_t) of ~3.3 min (β-(1→4)-isomer elutes earlier). Due to lack of standards, the linkage type of GlcAra-2AB could not be unambiguously confirmed by MS/MS, but is assumed to be β-(1→2)-linked originating from a β-(1→3)-Glc unit, analogous to the β-glucosidase resistant, confirmed Glcβ(1→3)Glc-2AB.

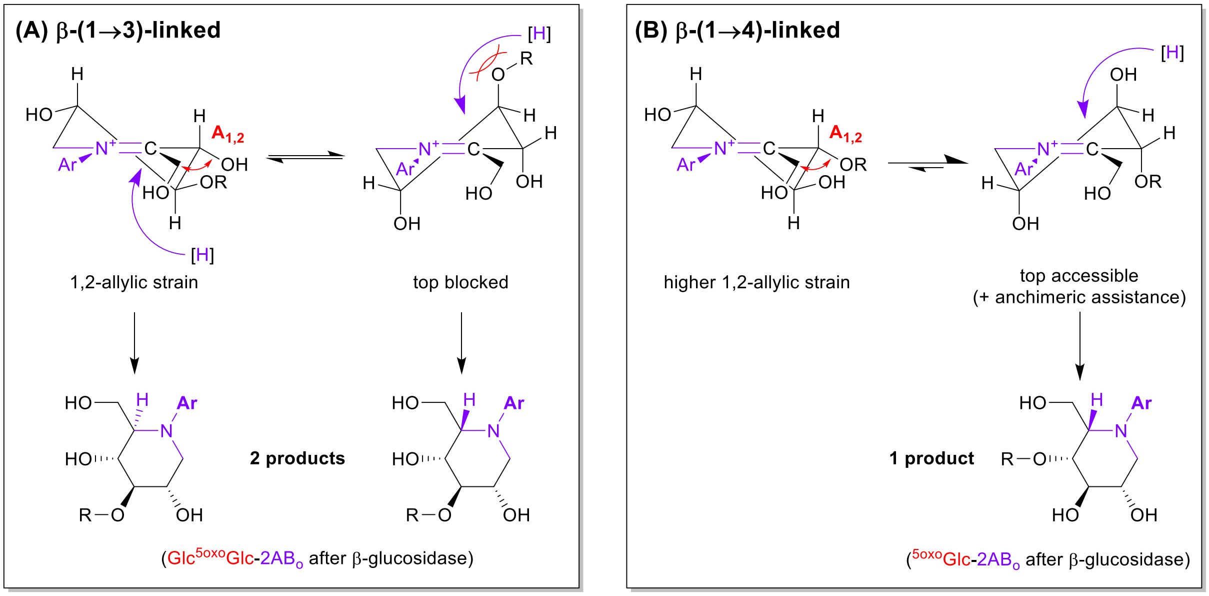

Figure S7 Detection of lytic C5-oxidation products with C=O labeling strategy III (reductive amination):[1] Proposed mechanisms to explain the observed epimeric mixture of Glc^{5oxo}Glc-2AB$_o$ (2 peaks), but not for Glc^{5oxo}Glc-2AB$_o$ (predominantly 1 peak), on the basis of observations made by Baxter and Reitz (1994) in their aza-sugar synthesis from 5-oxo-hexoses (see **Figure S8a** for chromatogram, and **Figure 8** for the full mechanism). Under the assumption that the hydride attack ($NaBH_3CN$) on the intermediate iminium ion takes place axially from the side that avoids formation of a boat-conformation transition state, a **(A)** β-(1→3)-linked unit has disfavoring steric obstacles for both possible intermediates, namely 1,2-allylic strain ($A_{1,2}$; left) pushing the equilibrium to the right side, and a blocked top side from R = Glc$_m$ (right). Consequently, products from both intermediates are formed. **(B)** A β-(1→4)-linked unit has two factors favoring the conformation on the right-hand side: higher 1,2-allylic strain due to R = Glc$_m$ (left), and an accessible top that additionally might have a directing effect of the free hydroxyl group at C3 by anchimeric assistance (right). This would explain why predominantly one product was formed for ^{5oxo}Glc-2AB$_o$ (β-(1→4)-linked before β-glucosidase), while both epimers were detected in comparable amounts for β-(1→3)-linked Glc^{5oxo}Glc-2AB$_o$. The high dependence on selectivity regarding substitution pattern and configuration was also observed by Baxter and Reitz (1994), as for instance unsubstituted 5-oxo-glucose gave high selectivity (>95%) for one epimer after reductive amination, while mannose (C2 epimer of glucose) and per-*O*-acylated 5-oxo-Glc had low selectivity (67:33 and ~50:50, respectively).

[1] ^{5oxo}Glc is the only primary oxidation product with 6 carbons expected to result in such a cyclization: For instance, a γ-keto-aldehyde (C4-oxidation) could also lead to a cyclisation by reductive amination resulting in a 5-membered pyrrolidine derivative with the same *m/z*. However, the C4-oxidation would have to occur on a reducing end for C1 to be a free aldehyde (in equilibrium with its hemiacetal form), and reducing ends are in low amounts compared to the total sugar units, most of which (>99%) are mid-chain units. Lytic C5-oxidation is the only process that directly leads to a suitable substrate for the observed cyclisation without the need for two oxidation processes happening on the same glucose unit. Misidentification of dehydration side reactions can also be excluded: A loss of 18 Da corresponds to -H_2O, or a dehydration, but cannot be a side product of Glc reducing end labeling, as test reactions with glucose and oligomer standards under identical reductive amination conditions showed no such products. It also cannot be a result of lactone formation with the carboxyl of the label, as this would lead to the correct *m/z* for 2-AA (-H_2O), but not for 2-AB (-NH_3; would give the same *m/z* of 282.10 as ^{5oxo}Glc-2AA, which was not observed).

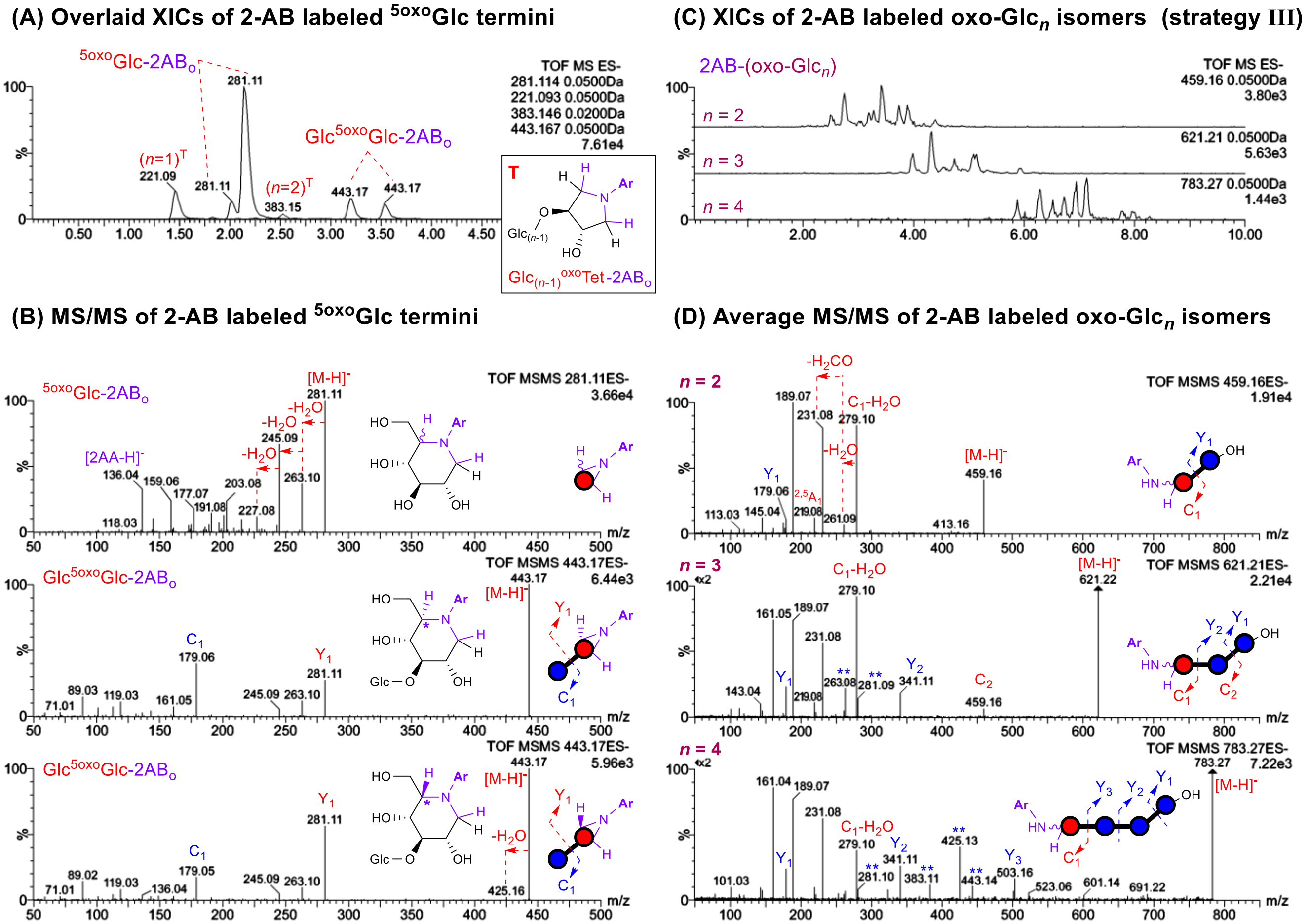

Figure S8 UPLC-MS/MS of oxo-products from BBG oxidation (harsh conditions) detected as 2-AB labeled species in SPE fraction 1 after reductive amination, enzyme treatment & SPE (strategy III; negative ion mode, basic eluent). **(A)** Overlaid extracted ion chromatogram (XIC) and **(B)** MS/MS spectra of C=O labeled 5-oxo-reducing ends (stereocenter * of epimers set arbitrarily). The inset labeled with "T" is the structure of 2-AB labeled L-*threo*-tetrodialdose (^{oxo}Tet-2AB$_o$), which is also a C5-oxidation product that was also observed by Schuchmann & von Sonntag in their Glc irradiation study (Schuchmann and Sonntag, 1977). **(C)** XICs of labeled oxo-Glc$_n$ products and **(D)** their respective MS/MS spectra. For each *n*, the average MS/MS is shown, as surprisingly little differences were found between the isobaric individual peaks of 2AB-(oxo-Glc$_n$) resolved by UPLC-MS.[2] The fragments are labeled assuming the labeled oxo-group being at the non-reducing end as in the depicted structures (since they are the main isomers as detected in strategy II; see **Figure 5a**).[3] Fragments labels in red contain the oxidized unit (incl. 2-AB), while labels in blue do not.

---

[2] It is noteworthy that in the MS/MS of these labeled reducing ends, some fragments in the spectra bear *m/z* that are easily mistaken to be purely glucose-derived fragments, but can be differentiated thanks to the qToF detection with high enough resolution (e.g. *m/z* 161.07 = [2AB·C_2H_3 – H]$^-$ vs. *m/z* 161.04 = [Glc – H_2O – H]$^-$). In some cases, this can be relevant as some cross-ring fragments (e.g. *m/z* 263.08, 281.09) which are indicative of the glycosidic linkage type are near-isobaric to 2-AB-labeled glucose fragments (e.g. *m/z* 263.10, 281.11). This also applies to MS/MS fragments in **Figure S6**.

[3] However, Y-ions are not typically encountered if the reducing end is still intact, as fragmentation in the negative mode predominantly occurs from the reducing to the non-reducing end link a zipper (A, B, & C ions). Hence, these ions, including the cross-ring fragments labeled with **, originate most likely from oxo-Glc$_n$ isomers with labeled mid-chain oxo-groups and are actually their C-type ions.

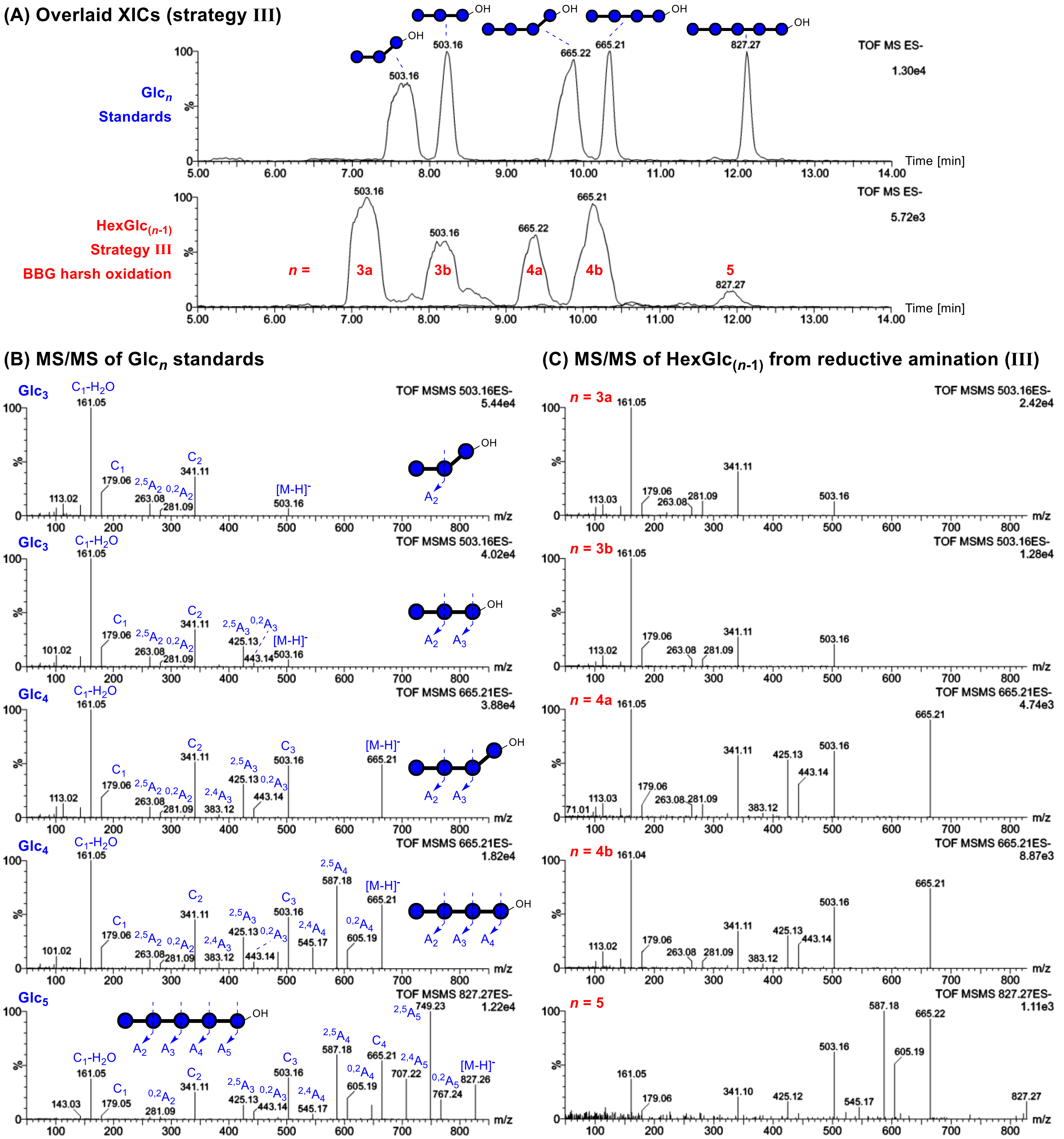

Figure S9 Comparison of **(A)** extracted ion chromatograms (XIC) from negative ion UPLC-MS (0.1% NH_3 eluent) and MS/MS of **(B)** Glc$_n$ standards (mixed-linkage & cello-oligomers) with **(C)** isobaric oligosaccharides formed under the reductive amination conditions during C=O labeling (strategy III, SPE fraction 1) of oxidized BBG (100 mM H_2O_2). The latter oligomers are presumably the result of direct C=O-reduction (instead of imin-reduction) of oxo-Glc$_n$ species with $NaBH_3CN$ to epimeric mixtures of HexGlc$_{(n\text{-}1)}$ (Hex = any hexose, e.g. glucose & its epimers (epimeric center in parenthesis) mannose (C2), allose (C3), galactose (C4); not necessarily at the non-reducing end). Note that retention times differ clearly from the standards in (A), and that all HexGlc$_{(n\text{-}1)}$ have a β-(1→3)-linked reducing end unit as evident in their MS/MS (C) (no A$_n$ ions).

MS/MS of CO_2H labeled standards and acidic oxidation products from BBG oxidation (strategy IV)

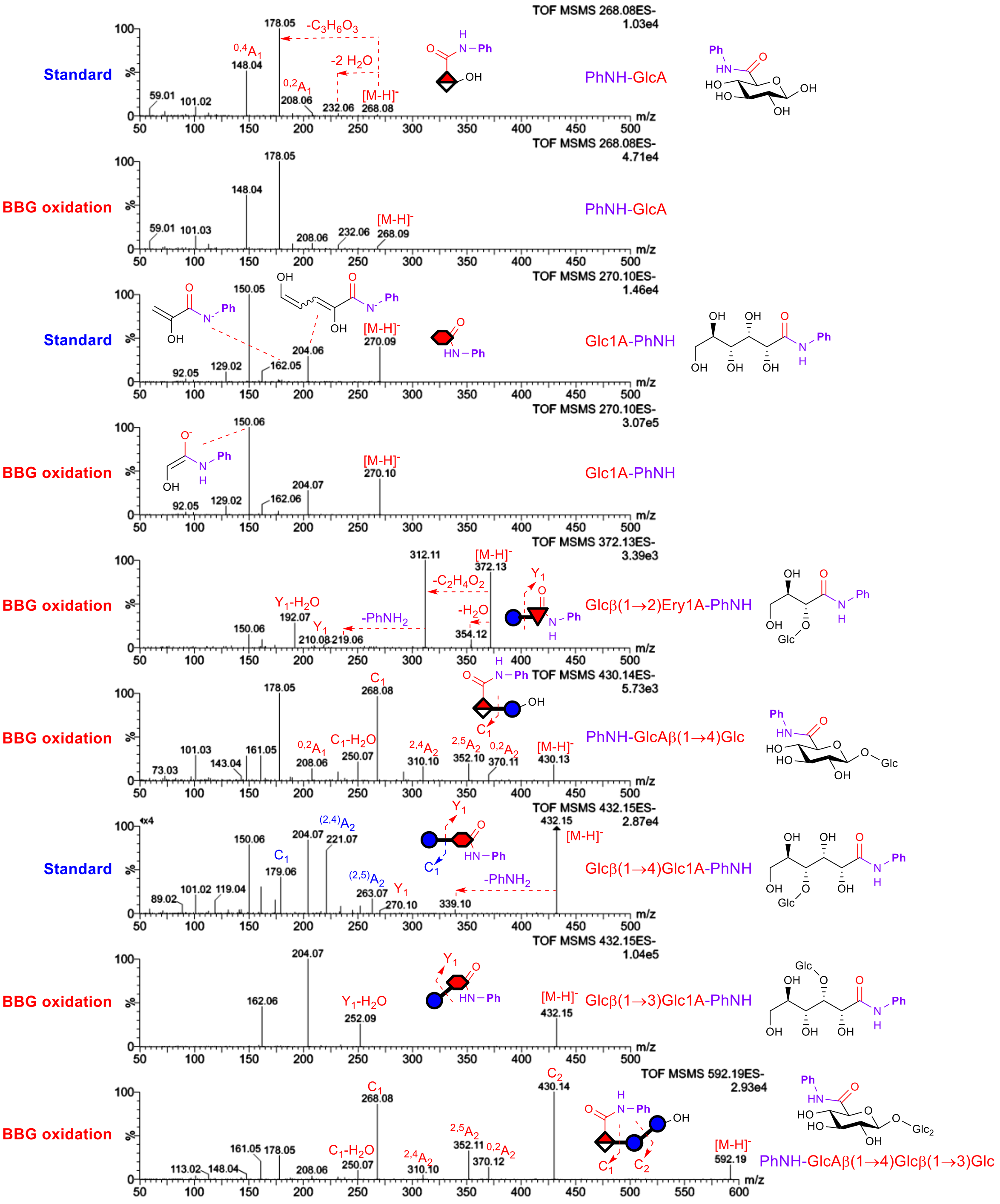

Figure S10 MS/MS spectra of CO_2H labeled standards and oxidation products from EDC-mediated amidation of carboxylic acids with $PhNH_2$ (sample preparation strategy IV). Note that the observed cross-ring fragments (A-ions) of labeled GlcAGlc (*m/z* 430) indicate a β-(1→4)-linkage, which is unexpected and might be a peeling product of $GlcAGlc_2$ (bottom spectrum).

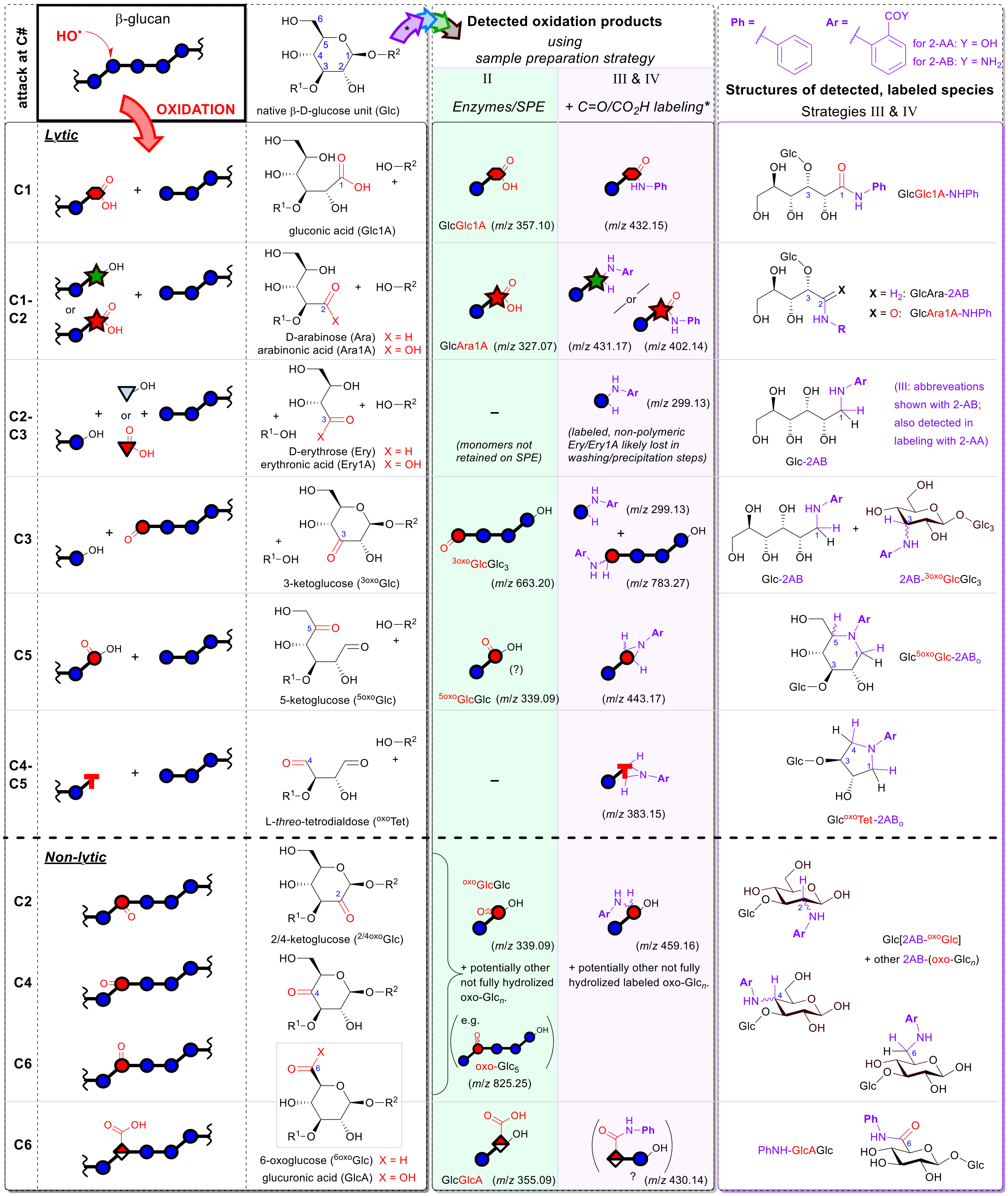

Figure S11 Symbols, structures and abbreviations: Lytic & non-lytic products initiated by HO$^{\bullet}$-attack on indicated carbons of β-glucan repeating unit (β-(1→3)-linked as example), as well as the resulting UPLC-MS detected products after enzyme treatment/SPE (strategy II) and with prior C=O/CO_2H labeling (strategy III & IV). Attack on a β-(1→4)-linked unit gives the analogous products (attack on C4 being lytic, on C3 non-lytic), but different sizes of detected products after sample preparation strategies II – IV (e.g. monosaccharides instead of Glcβ(1→3)[oxidized/labeled unit]). Cross-ring cleavages C1-C2, C2-C3 & C4-C5 according to Schuchmann and Sonntag (1977).

Table S1 Sample preparation strategies of oxidized BG solutions and the resulting lost and preserved information about the oxidation products for each case, as well as the corresponding species detected by UPLC-MS and confirmed by MS/MS.[a]

**Sample preparation**[b]	**Lost information**[c]	**Preserved information**	**Detected as**[d]
**I**: SPE	Polymeric oxidation products	Released oligosaccharides (neutral & acidic) with n = 2–8	(oxo-)Glc$_n$, Glc$_{(n\text{-}1)}$Ara, Glc$_{(n\text{-}1)}$Ery, Glc$_{(n\text{-}1)}$GlcA, Glc$_{(n\text{-}1)}$Glc1A, GlcAGlc$_{(n\text{-}2)}$Glc1A GlcAGlc$_{(n\text{-}2)}$Ara[h]
**II**: Lichenase + β-glucosidase, SPE	Neutral (new) reducing ends, lytic C1-oxidation[g]	(lytic) oxo-products, C6-oxidation to CO_2H, β-(1→3)-linked C1-oxidation products	^{oxo}GlcGlc$_{(n\text{-}1)}$, GlcAGlc$_{(n\text{-}1)}$, GlcGlc1A, oxo-Glc$_n$[i]
**III**: Carbonyl (C=O) labeling, precipitation, enzymes, SPE[e]	(lytic) oxo-products (e.g. from lytic C3/C4-oxidation),[j] acidic products	Neutral (new) reducing ends (incl. cross-ring cleavage products & lytic C5-oxidation)	(Glc)Glc-2AB, (Glc)Ara-2AB, (Glc)Ery-2AB, (Glc)^{5oxo}Glc-2AB
**IV**: Carboxylic acid (CO_2H) labeling, precipitation, enzymes, SPE[f]	Neutral (new) reducing ends, (lytic) oxo-products	C1- and C6-oxidation to CO_2H, acidic products from cross-ring cleavage	(Glc)Glc1A-NHPh, (Glc)GlcA-NHPh, PhNH-GlcAGlc$_{(n\text{-}1)}$, Ara1A-NHPh, GlcEry1A-NHPh

[a] Refers to harsh oxidation conditions used for method development (0.6% BG, 50 µM $FeSO_4$, 100 mM H_2O_2). Lost or preserved information as well as the detected products in the case of the two labeling procedures (III & IV) refer to only the respective labeled products. oxo-Glc$_n$, gluco-oligomer with an oxidized hydroxyl group (→C=O) on any of the units; ^{oxo}GlcGlc$_{(n\text{-}1)}$, gluco-oligomer with carbonyl specifically at the non-reducing end unit; -2AB, reducing end unit labeled with 2-AB by reductive amination (→aminodeoxyalditol); -NHPh or PhNH-, anilide of acid unit. For other abbreviations and structures, see **Figure S11**.

[b] Oxidized BG solution treated with phosphate buffer and catalase first (except for strategy I: SPE).

[c] Monosaccharide and other small products (<C_6) are lost in all cases due to SPE purification/fractionation.

[d] (Glc) and (oxo-) in parenthesis refer to products both with and without the indicated additional structural feature.

[e] Labeling by reductive amination with 2-AB (as example, 2-AA also possible) and $NaBH_3CN$. Enzymes: lichenase + β-glucosidase treatment.

[f] Labeling by amidation with aniline ($PhNH_2$) and EDC. Enzymes: lichenase + β-glucosidase treatment.

[g] Information only partially lost, namely β-(1→4)-linked C1-oxidation products.

[h] Mixed-linkage mixtures with random position of both β-(1→3)-linkage and oxidized sugar unit (e.g. GlcA, C=O group).

[i] The predominant products exhibit clearly defined position of the β-(1→3)-linkage (reducing end unit) and the oxidized monomer unit (as the non-reducing end; n = 2–5). Prominent exceptions are Glcβ(1→3)Glc1A & Glcβ(1→3)GlcA, as well as some oxo-Glc$_n$ products (n = 2, 4, 5).

[j] Partially lost through direct C=O reduction to epimeric mixtures HexGlc$_{(n\text{-}1)}$ as side-reaction of reductive amination.

References

Baxter, E.W., and Reitz, A.B. (1994). Expeditious Synthesis of Aza sugars by the Double Reductive Amination of Dicarbonyl Sugars. *The Journal of Organic Chemistry* 59(11)**,** 3175-3185. doi: 10.1021/jo00090a040.

Schuchmann, M.N., and Sonntag, C.V. (1977). Radiation-Chemistry of Carbohydrates .14. Hydroxyl Radical Induced Oxidation of D-Glucose in Oxygenated Aqueous-Solution. *J. Chem. Soc. Perk. T. 2* (14)**,** 1958-1963. doi: 10.1039/P29770001958.
